# Silica-Based Gene Delivery Systems: From Design to Therapeutic Applications

**DOI:** 10.3390/pharmaceutics12070649

**Published:** 2020-07-09

**Authors:** Ana Maria Carvalho, Rosemeyre A. Cordeiro, Henrique Faneca

**Affiliations:** Center for Neuroscience and Cell Biology, University of Coimbra, 3004-504 Coimbra, Portugal; amcarv95@gmail.com (A.M.C.); rose.a.cordeiro@gmail.com (R.A.C.)

**Keywords:** silica-based vectors, hybrid silica nanosystems, silane chemistry, targeted gene delivery, stimuli-responsive release, gene therapy

## Abstract

Advances in gene therapy have been foreshadowing its potential for the treatment of a vast range of diseases involving genetic malfunctioning. However, its therapeutic efficiency and successful outcome are highly dependent on the development of the ideal gene delivery system. On that matter, silica-based vectors have diverted some attention from viral and other types of non-viral vectors due to their increased safety, easily modifiable structure and surface, high stability, and cost-effectiveness. The versatility of silane chemistry and the combination of silica with other materials, such as polymers, lipids, or inorganic particles, has resulted in the development of carriers with great loading capacities, ability to effectively protect and bind genetic material, targeted delivery, and stimuli-responsive release of cargos. Promising results have been obtained both in vitro and in vivo using these nanosystems as multifunctional platforms in different potential therapeutic areas, such as cancer or brain therapies, sometimes combined with imaging functions. Herein, the current advances in silica-based systems designed for gene therapy are reviewed, including their main properties, fabrication methods, surface modifications, and potential therapeutic applications.

## 1. Introduction

In recent decades, gene therapy advances have paved the way towards the effective treatment of inherited and acquired diseases involving genetic factors. By modifying abnormal and/or introducing gene sequences, gene therapy can correct the pathophysiology at the gene expression level, treating diseases such as cancers, Parkinson’s disease, cardiovascular diseases or acquired immunodeficiency syndrome (AIDS) [1,2,3].

**Figure 1:**
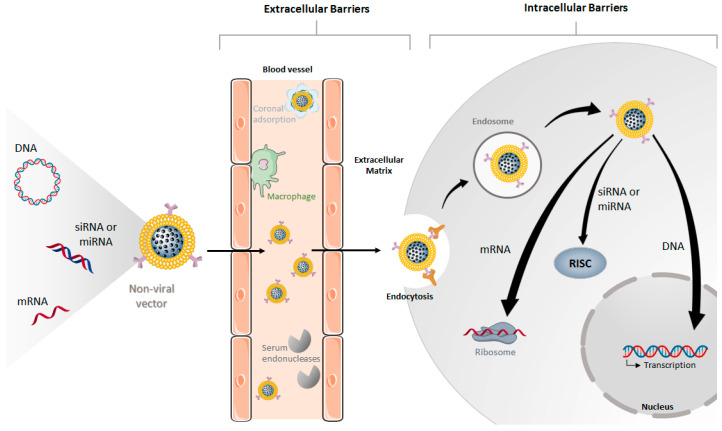
Physiological barriers to systemic delivery of different types of nucleic acids using non-viral vectors. Extracellular barriers include degradation by endonucleases, adsorption of serum proteins, clearance by the renal system or reticuloendothelial system (RES), and extracellular matrix penetration. After targeted cellular uptake, endo/lysosomal escape is also required for successful delivery. Finally, siRNA and miRNA molecules must be loaded into the RNA-induced silencing complex (RISC), while mRNA must be read by the ribosome and DNA ought to enter the nucleus for transcription.

Naked genetic material molecules, besides not having the ability to target specific cells and be internalized by them, are not able to successfully reach a target tissue before being degraded by serum endonucleases [2,4]. Thus, a crucial factor in determining the success of gene therapy is the development of safe and efficient gene delivery systems capable of protecting the genetic material and overcoming the critical physiological barriers to gene delivery (Figure 1), while increasing the transfection efficiency and specificity [4,5]. Ideally, these carriers should introduce targeted gene delivery properties with controlled release kinetics, to reduce the undesired off-site effects, and thus improve the effectiveness of the treatment [1,2,6].

For the delivery of nucleic acids into target cells, two main types of vectors have been used: viral and nonviral. Viral delivery systems have been extensively studied in clinical trials for gene therapy purposes, and products based on viral vectors have been approved by the European Medical Agency (EMA) and the Food and Drug Administration (FDA). However, these viral carriers inherited fundamental drawbacks, such as immunogenicity/pathogenicity, uncontrolled integration into the host genome, production issues, or cargo capacity [1,7]. For nonviral delivery of nucleic acids, different types of carriers, such as lipid-, polymer-, peptide-, or inorganic-based nanosystems have been used [1,2,7]. These systems overcome important limitations associated with viral vectors and are superior in terms of safety [1,2]. Among the inorganic materials used as gene carriers, silica-based nanosystems have drawn some attention due to their properties: these are free of some of the limitations of organic nanosystems, such as their low stability in physiological conditions, and offer many advantages when compared with other types of inorganic materials, such as controllable size and easily modifying surface, can be conveniently produced in a large scale, and more importantly, have great biocompatibility [3,8,9]. It is noteworthy that silica has been given the status of “Generally Recognized as Safe” by the FDA [10,11]. Furthermore, the first silica-based nanoparticulate system, in the form of Cornell dots (“C-dots”) [12,13], has recently received FDA approval for Phase-I clinical trial, for targeted molecular imaging, and no toxic or adverse events related to the particles were observed [14]. This has given significant support to the clinical viability of silica-based nanosystems, providing a boost in research in this direction. In this sense, several different types of silica-based structures have been envisioned and studied not only for gene delivery purposes but also for other biomedical applications such as imaging or drug delivery, having silica nanoparticles appeared in some clinical trials in the last decade (Table 1) [10,15,16,17].

The properties of any nanosystem, such as its size or shape, play a major role in its in vivo performance, and therefore, when delivering genetic material, these properties should be carefully tailored in order to enhance the system’s cellular uptake and transfection efficiency. The easily modifiable synthesis of silica-based structures from silane precursors allows the control of these functional properties and even grants the possibility of incorporating different stimuli-responsive organic groups into the silica framework, which can confer the nanosystem with triggered biodegradation properties for the controlled release of payloads [18,19]. Regardless of the system’s core-design and structure, another very important aspect to account for in any silica-based gene delivery system is its surface functionalization [20,21]. Bare silica materials acquire a negative surface charge in aqueous conditions due to the presence of silanol groups on their surface. Therefore, to achieve efficient gene delivery, functionalization of the surfaces is usually done with cationic groups, enabling the systems to condense and load DNA through electrostatic interactions with the negative charges of nucleic acids’ phosphate backbone [22,23]. This functionalization is also important to enhance the cellular uptake of the system since the resting potential of the cell membranes is also negative [24]. A simple and common attempt to enhance gene loading capacity of silica materials is amination modification with trialkoxysilanes such as (3-aminopropyl)triethoxysilane (APTES) or (3-aminopropyl)trimethoxysilane (APTMS) [20]. However, in more complex systems, these silanes can be used as linkers for the grafting of other types of components, such as antibodies, peptides, or polymers, which form a coating shell capable of fulfilling different roles in the nanosystem’s in vivo performance, being the use of polymers the most commonly explored and reported strategy [16].

**Figure 2:**
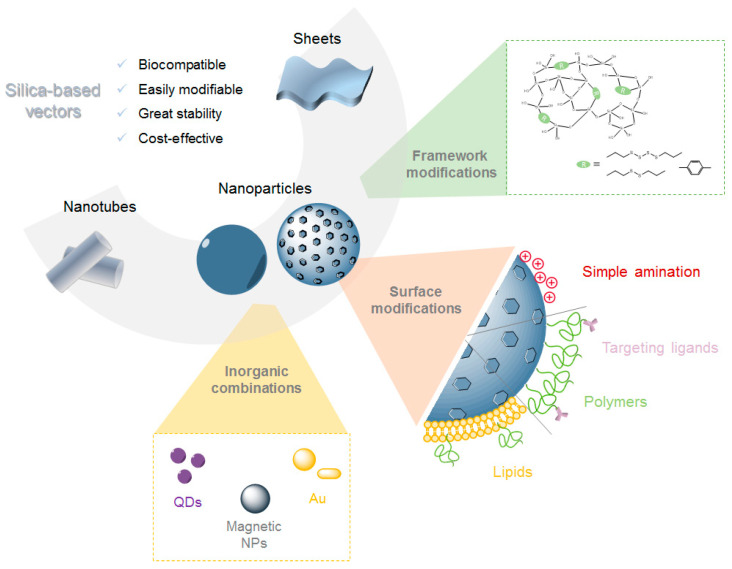
Different types of silica-based vectors reported for gene therapy and discussed in the present review.

This review covers the different types of silica-based structures (Figure 2) that have been recently reported for gene delivery applications, such as silica nanotubes, silica sheets, and a variety of nanoparticles-based systems, including their main properties, fabrication methods, surface modifications, and potential therapeutic applications.

## 2. Silica Nanotubes

**Figure 3:**
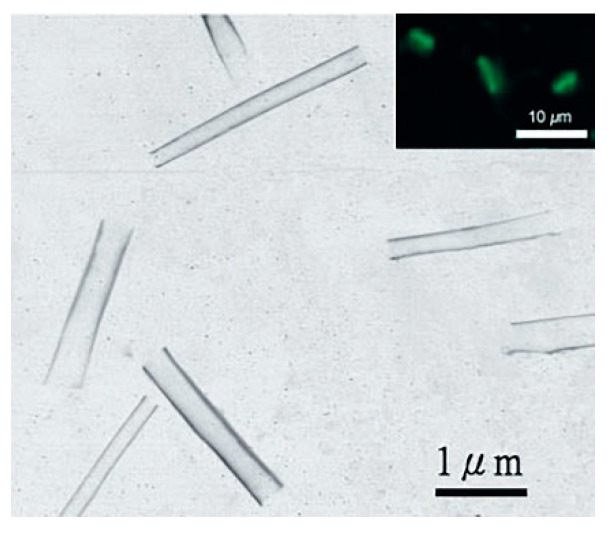
Transmission Electron Microscopy (TEM) and fluorescent images of fluorescent SNTs. Reproduced from [22], with permission from John Wiley and Sons.

Silica nanotubes (SNTs) have become promising carriers for biomedical applications, due to their unique properties. Unlike nanoparticles or nanorods, SNTs are cylindrical nanostructures with monodisperse diameters and a hollow structure which grants them two physically different domains: an inner void and the outer surface (Figure 3) [25]. The inner space can be filled with specific biomolecules, which are conveniently shielded by the tube structure. Additionally, the differential functionalization of the inner and outer surfaces could also allow the straightforward development of a multifunctional system [22,23,25].

The most common approach for synthesizing SNTs is the template method, which entails the synthesis or deposition of the material within the cylindrical nanopores of a template membrane that can be dissolved away afterward [9,25]. This method is very popular, since the properties of tubular structures are well directed by the template and can be easily customized by adjusting the template structure [26]. It has been used to prepare solid (nanorods or nanowires) and hollow structures (nanotubes) composed not only of silica but also different types of materials, such as polymers or carbon [25]. Anodic aluminum oxide [22,23] and collagen [26] membranes have been used as templates, on which a silica layer is deposited through sol–gel reactions [9]. Building upon this concept, Yu and colleagues [27] developed a mechanical capping method of SNTs using an alumina microbead hammering treatment, which could be used to encapsulate functional molecules into the nanotubes. It was suggested that not only inorganic metals but also polymers can be used as caps for the SNTs, using this method.

**Figure 4:**
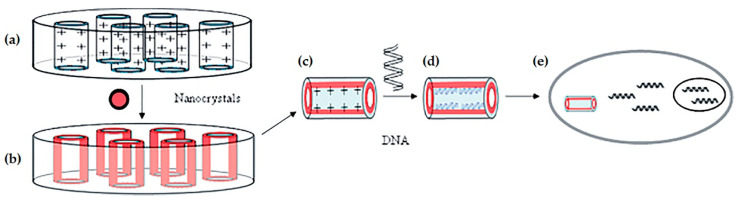
Schematic illustration of the fluorescent SNTs preparation and its application in gene delivery: (**a**) The anodic Al_2_O_3_ membrane coated with a silica layer is modified with APTMS to form a cationic surface; (**b**) Nanocrystals are incorporated onto the polycationic surface. An additional silica layer and a subsequent APTMS coating are layered over the nanocrystals; (**c**) The SNTs with polycationic inner surfaces are generated by removal of the membrane; (**d**) pDNA is complexed into the nanotubes; (**e**) The complexed system enters the cell and the DNA is released and transcribed in the nucleus. Reproduced from [22], with permission from John Wiley and Sons.

Despite their advantages, the use of SNTs as gene carriers is still quite rare and not heavily reported. In 2005, Chen and co-workers were the first to introduce SNTs as a gene delivery system [22]. They synthesized fluorescent silica nanotubes through a sol–gel reaction by incorporating CdSe/ZnS nanocrystals and using an alumina membrane as a template (Figure 4). Afterward, the inner surface of the nanotubes was functionalized with APTMS, creating a cationic surface capable of efficiently condense and load DNA. According to confocal microscopy results, 60 to 70% of cultured mammalian cells (COS-7) internalized the nanotubes during incubation, which also proved to be non-cytotoxic. Moreover, the SNT/DNA complexes successfully transfected 10–20% of the treated cells, opposite to the absence of transfection registered with the free DNA. In this work, due to the concomitant observation of plasmid DNA (pDNA) and nanotubes inside the cells, the plasmid delivery was suggested to be mediated by the nanotubes, which proved to act as a physical shield, preventing the genetic material degradation.

Later on, Namgung and colleagues designed a different SNT-based system, using branched polyethylenimine (bPEI) as a complexing agent, which is generally accepted as the gold standard for polymer-based gene delivery vectors, presenting high transfection efficiency [23]. Contrary to the vector developed by Chen and co-workers [22], in Namgung’s system [23], the outer surface of the SNTs was covalently functionalized with bPEI, complexing the DNA outside the tubes, while the inner space of the SNTs was filled with iron oxide nanoparticles and green fluorescent quantum dots. These two cargos allow to simultaneously study the nanotubes by magnetic resonance imaging (MRI) and monitor their intracellular movement by fluorescence, resulting in a multifunctional nanosystem. The use of a low molecular weight (LMW) bPEI (1.8 kDa) allowed a large accommodation of the polymer in the SNT, therefore acquiring a high charge density capable of successfully complex and compact pDNA. At a nitrogen/phosphate (N/P) ratio of 10, the generated carriers transfected more than 94% of the cells. Additionally, at the same N/P ratio, the system showed a much higher transfection efficiency than polyplexes based on LMW bPEI and similar results to those obtained with high molecular weight bPEI (the gold standard). Surprisingly, it was found that, despite the already mentioned negative charge of uncoated SNTs, they could load a small amount of pDNA on their surface through weak hydrogen bonding or Van der Waals interactions and enter the cells through a non-specific endocytic pathway [23,24]. Moreover, confocal fluorescence microscopy results revealed that SNTs existed only in the cells’ cytoplasm, while the genetic material was entirely localized in the nucleus within 4 h. On the other hand, the bPEI-coated SNTs were able to enter the nuclei, their movement being overlapped by that of pDNA [23].

The size of the nanotubes is a crucial characteristic that has been proved to have a great impact on their in vivo performance. Several studies using carbon nanotubes have shown that an efficient cellular uptake is attained with nanotubes smaller than 1 μm [28]. Chen and colleagues developed SNTs with controlled length ranging from less than 500 nm to almost 2 μm and their results proved that the cellular uptake of SNTs was length-dependent, increasing with the decrease of size. In this study, the outer surface of the developed nanotubes was functionalized with chitosan (ChSNT), another cationic polymer, which was used to deliver immunostimulatory cytosine-phosphodiester-guanine oligodeoxynucleotides (CpG ODNs). The developed system was found in the endolysosomes after cellular internalization and significantly enhanced both the cellular uptake and immunostimulatory response of CpG ODNs [26].

## 3. Silica Sheets

Gene delivery is commonly performed using forward transfection methods, i.e., procedures in which cell plating is done first and then genetic material is added using different types of vectors. However, reverse transfection methods have also been designed by adsorbing the reagents and plasmid DNA onto culture substrates before cell seeding [29].

**Figure 5:**
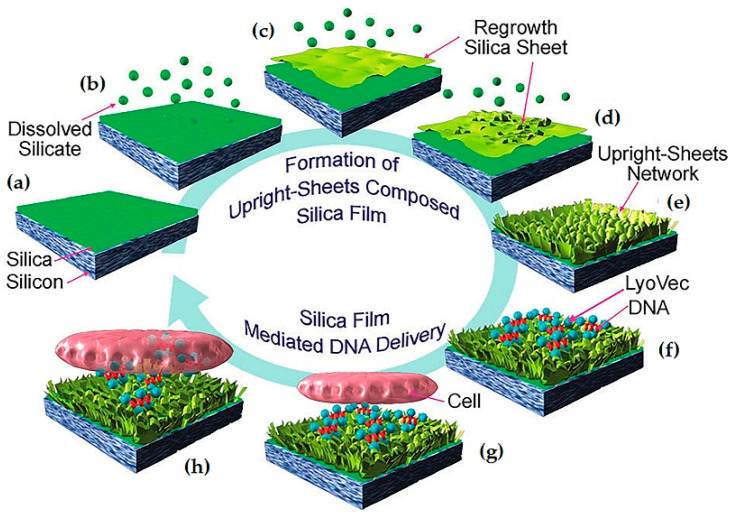
Illustrative scheme of the formation steps of an upright network of silica sheets (**a**–**e**), and film mediated DNA transfection to the cell (**f**–**h**) (reproduced from [30] with permission from The Royal Society of Chemistry).

Silica sheets are substrates that possess large surface areas and provide useful platforms for trapping or adsorbing biomolecules. Ji and colleagues were the first to report a method for reverse gene transfection using a silica film with an upright-sheet network [30]. This was carried out by a one-pot process that started with the sputtering of a silica layer and further incubation with an aqueous NaBH_4_ solution capable of etching that layer, leading to the appearance of long wrinkles and knobs until it transformed into a crosslinked network of silica nanosheets (Figure 5a–e). The surface of the nanosheets was additionally modified with APTES to allow the electrostatic immobilization of DNA, which the authors found to be much greater in regions with networks of upright sheets than in flat silica films. In this work, reverse transfection was performed on human embryonic kidney mammalian cell line (HEK293XL/null). The cells adhered to the silica substrate loaded with complexes of DNA and a lipid vector, as illustrated in Figure 5f–h, and a higher gene transfection was achieved on the dense upright sheet network. It was also reported that the transfection efficacy obtained by this reverse transfection protocol doubled that obtained by forward transfection, probably due to the direct contact between cells and the surface [30].

**Figure 6:**
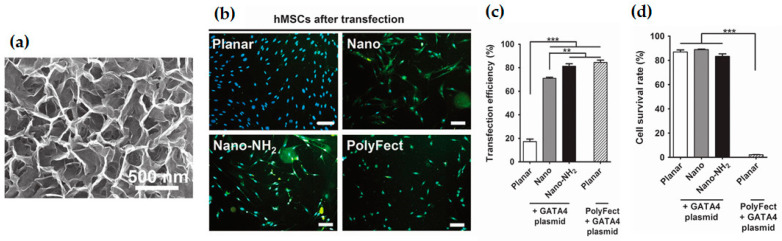
(**a**) SEM image of the silica upright nanosheet network; Fluorescent images (**b**) and the corresponding transfection efficiency (**c**) of human mesenchymal stem cells (hMSCs) after transfection with GATA4 plasmid on a planar silica surface (Planar), unmodified nanosheets (Nano), amino-modified nanosheets (Nano-NH_2_) and with the transfection agent PolyFect on the planar surface (Polyfect) (the scale bar represents 50 μm); (**d**) Cell survival rate of hMSCs, determined by flow cytometry, at 60 h post seeding (and 84 h in the Polyfect group). Reproduced from [31], with permission from Springer Nature.

Later on, Huang and co-workers [31] improved the fabrication method previously developed [30] and increased the yield of produced nanosheets using a simple and easy to scale up approach. The network of silica nanosheets was optimized for gene delivery and presented a pore diameter of 400 nm, an average wall thickness of 5 nm, and wall height of 120 nm (Figure 6a), being successfully used for the vector-free delivery of naked plasmid DNA into human stem cells and HEK cells. It was suggested that the delivery was achieved by an integrin cell signaling pathway which is activated by the network nano-topography. As formerly reported [30], their results also showed that the transfection efficiency was much greater for the cells plated in the amine-coated sheets network (~85%) than in planar silica (~20%), as presented in Figure 6b,c. Additionally, the vector-free delivery in upright silica-sheets proved to be more time-efficient (shorter by 24 h) and overall effective than the commercial transfection reagent, PolyFect, in planar silica, since it presented similar transfection efficiency but was much less cytotoxic (cell survival rate of 80–90%, compared to about 3% survival with PolyFect—Figure 6d) [31]. In a different study, the effect of several properties of the silica nanosheets on the transfection efficiency was also assessed [29]. By changing the reaction time and mixture of the silica layer chemical etching, silica networks with different geometric features and physicochemical properties were obtained. It was found that the surface morphology of the nanosheets had a greater influence on transfection efficiency than the surface zeta potential, hydrophilicity, or surface chemistry. More precisely, the aspect ratio (height/thickness of the wall) of the nanosheets was found to be the most critical factor positively influencing the cell migration rate and gene transfection efficiency on the nanosheets. Additionally, in a more recent study, the trapping capacity of a silica nanosheets structure was demonstrated to be almost solely related to its morphological features rather than with its surface functionality [32].

**Figure 7:**
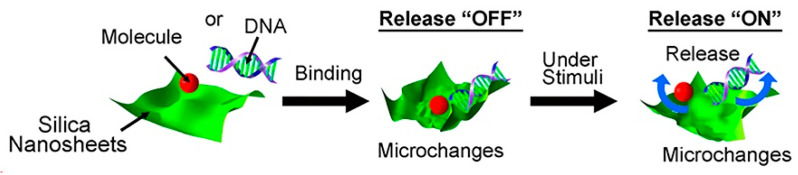
Graphic illustration of the encapsulation and stimuli-responsive release of biomolecules by silica nanosheets. Adapted with permission from [32]. Copyright (2017) American Chemical Society.

More recently, a loose aggregate of silica nanosheets has been developed, forming a sponge-like porous structure. The encapsulation and immobilization of molecules inside this structure was possible due to the void space inside the pores, as well as the wrapping effect of the nanosheets. Additionally, the “softness” and morphological flexibility of the structure enhanced its encapsulation properties and granted it a sustained and stimuli-responsive release behavior, which could be triggered by external stimuli, such as sonication or treatment with a buffer solution, as illustrated in Figure 7. Immobilized DNA molecules were successfully released in response to the nanosheets structure rearrangement promoted by the application of PBS solution [32].

## 4. Silica Nanoparticles

Silica nanoparticles (NPs) can be categorized into two main groups: solid (non-porous) silica NPs and porous silica NPs. The latter can be further subdivided according to the size of their pores, being mesoporous silica nanoparticles (MSNs) the most studied category among porous silica NPs. Recent advances in the use of non-porous silica NPs and MSNs as gene carriers are described in the sections below.

### 4.1. Non-Porous Silica Nanoparticles

Several synthetic techniques have been developed to produce homogeneous silica nanoparticles, but the two most commonly used ones are reverse microemulsion and the Stöber method. Briefly, the reverse microemulsion, or water-in-oil (w/o) system, comprises a homogeneous mixture of water, oil, and surfactant molecules that form reverse micelles. The aqueous phase of the system is then confined to the bulk of the micelles, acting as a nanoreactor for particle formation [18]. The Stöber method, developed in 1968, is a typical sol–gel process in which a solution gradually evolves towards a gel-like network [33]. In this process, the nanoparticles are formed by the hydrolysis and condensation of a siloxane precursor, such as tetraethyl orthosilicate (TEOS), using water and alcohol as solvents, and ammonia as a catalyst. Despite all the advances in this area, the classical Stöber method continues to be one of the most widely used methods to synthesize monodisperse solid silica NPs with size ranging from 5 to 2000 nm.

As previously mentioned, pure silica NPs without surface modifications, present a negative zeta potential that hinders electrostatic interaction with the negatively charged nucleic acid molecules [34]. However, in the early 2000s, the Saltzman’s group reported that unmodified silica NPs were able to enhance gene delivery in cultured cells by helping other cationic transfection reagents, acting as a sedimentation agent, that is, the dense silica NPs worked by concentrating the complexes of DNA-transfection reagent at the surface of cells due to gravity [35,36,37]. The increase in transfection efficiency when using silica NPs has also been reported by Guo and co-workers; however, they suggested that the NPs acted as more than just a sedimentation agent, operating also as a secondary transfection mediator [38]. These findings increased the interest in the application of silica NPs for gene delivery but nowadays most of their reported usages encompass surface modifications.

Lehr’s group was the first to use functionalized silica NPs for gene delivery [39,40]. They covalently linked trialkoxysilanes with amine groups to the surface of commercially available silica NPs and proved their ability to electrostatically bind, condense and protect plasmid DNA, forming a complex capable of successful in vitro transfection. By incubating the cells with 100 μL of transfection medium supplemented with 100 μM of chloroquine, the transfection efficiency of the silica NPs-DNA complex reached 30% of that achieved with PEI-DNA polyplexes and no cytotoxicity was observed for the modified NPs at those concentrations, while 50% cytotoxicity was observed with PEI [40]. Amine-coated silica NPs were also tested for their in vivo transfection efficiency in DBA/2 mice lung cells, and a two-fold increase in the expression levels of GFP was observed in comparison to plasmid encoding enhanced GFP alone. Additionally, almost no cytotoxicity was observed, highlighting their potential as gene carriers [41].

Since then, numerous other studies have been reported using silica NPs with amine groups at the surface for gene delivery purposes [34,42,43,44]. Bharali and colleagues studied the performance of amino-functionalized silica NPs as gene delivery systems for in vivo delivery to the brain of mice via stereotaxic injection [42]. They proved that the modified NPs were able to bind and protect plasmid DNA from enzymatic degradation and obtain a transfection efficiency equal or higher than that obtained using HSV-1 viral vectors. Moreover, no toxicity was observed with silica NPs even 4 weeks after transfection, while tissue damage was observed using the viral vector due its toxicity and immunological side effects [42]. Huang’s group developed amino-modified silica NPs to use as vectors for hepatocellular carcinoma gene therapy and proved their efficiency in vitro and in vivo [45]. The nanoparticles were used to complex a p53-encoding plasmid DNA and to transfect HepG2 cells with high efficiency and reduced cytotoxicity. Moreover, their injection in tumor-bearing mice resulted in significant tumor growth inhibition [45]. The p53 plasmid was also delivered by luminescent silica NPs, developed by Chandrababu Rejeeth and co-workers, in breast cancer models, both in vitro and in vivo [44]. In this study, the silica NPs were synthesized through a microemulsion technique and further functionalized with amine groups. The luminescent silica NPs showed a higher transfection efficiency than a commercial transfection reagent (Lipofectin^®^) in MCF-7 cells. Additionally, the results obtained in the PCR analyses demonstrated that the nanoparticles were able to maintain the integrity of the plasmid DNA in serum, and to extend the mRNA expression of the plasmid for up to 5 days in various organs [44]. More recently, Reinhardt and colleagues reported the production of novel silica NPs exposing quaternary ammonium groups at the surface by performing a surface modification with amino-silanes and further methylation of the primary and secondary amine [43]. Compared to non-quaternized amine-modified NPs, the quaternary ammoniums imparted the nanoparticles with great colloidal stability and DNA binding effectivity, particularly at high salt concentrations. Moreover, they studied the electrostatic interactions of the developed nanosystem with cationic liposomes and were able to form a novel type of ternary assembly where DNA strands were sandwiched between the NPs modified surface and a cationic lipid bilayer [43]. 

Besides their surface properties, the nanoparticles’ size also has a great influence on their in vivo performance. Chengzhong’s group synthesized monodisperse silica NPs with amine-modified surfaces and diameters ranging from 125 to 570 nm and studied their transfection efficiency in HEK293Y cells [46]. They found that the transfection efficiency was a compromise between the NPs binding capacity and their cellular uptake, the best result being achieved with 330 nm nanoparticles [46].

### 4.2. Porous: Mesoporous Silica Nanoparticles

MSNs are characterized by the presence of pores with diameters between 2 and 50 nm throughout their structure, which grants them large pore volumes, high surface areas, and a much larger surface-to-volume ratio than solid NPs. In the last few decades, different types of silica architectures have been explored to achieve attractive physicochemical properties for different applications [11]. 

**Figure 8:**
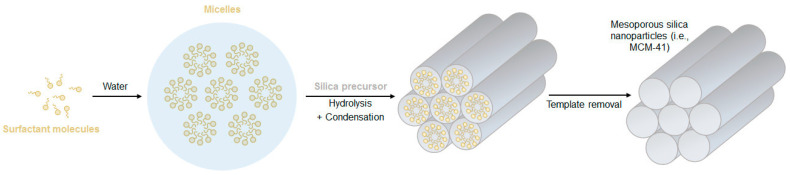
Schematic illustration of the formation mechanism of mesoporous silica nanoparticles (MSNs).

Generally, MSNs are synthesized through modified Stöber processes, which encompass the addition of pore templating agents, such as surfactants or polymers. Typically, the surfactant is stirred in a mixture of water and alcohol, under basic conditions, and if its concentration is above the critical micelle concentration, it self-assembles into micelles that will work as pore templates. As illustrated in Figure 8, when the silica precursor is added to the solution, it condensates at the surface of the existing micelles, forming a silica structure around them. To complete the process, the surfactant micelles are removed from the silica structure through high-temperature processing (calcination) or by solvent extraction. Using this method, the obtained pore size is usually very uniform and easily tunable by varying the type of surfactant used, but it commonly ranges between 2 and 5 nm [8,11,18].

Using cetyltrimethylammonium bromide (CTAB) as a pore template surfactant, Chang and colleagues developed FITC-conjugated MSNs with a hexagonal shape and uniform size of approximately 110 nm, with an average pore size of 2 nm [47]. To successfully conjugate these nanoparticles with negatively charged genetic material, they were positively charged by surface functionalization with *N*-triaminethoxysilylpropyl-*N*,*N*,*N*-trimethylammonium (TMA) chloride. These MSNs were successfully used to co-deliver a plasmid DNA encoding Nurr1 (pNurr1) and siRNAs against Rex1 (siRex1) to induced pluripotent stem cells (iPSCs) to promote their differentiation into dopaminergic neurons, which was confirmed by the detection of dopamine secretion. In addition to the great cell uptake efficiency of the nanosystem (up to 80%), it also proved to be more biocompatible than the commercial transfection reagent Lipofectamine^®^ 2000. Moreover, the authors of this work also demonstrated that the co-delivery of the two types of nucleic acids (pNurr1 and siRex1) presented a synergistic effect, resulting in the enhancement of Nurr1 gene expression by three-fold when compared to the single delivery of pDNA [47].

Furthermore, recent investigations have used MSNs to explore the combination of gene therapy with chemotherapy, aiming to overcome drug resistance and improve the therapeutic outcome by co-administrating drugs and genetic material [10]. For instance, in a recent study, Bcl-2 siRNAs were connected to MSNs surface via disulfide linkers, acting as redox-responsive gatekeepers of DOX loaded inside the mesopores [48]. The system successfully escaped lysosomal degradation and was able to inhibit the expression of Bcl-2 protein. Additionally, it was found that the co-delivery of DOX and siRNA with this nanocarrier displayed synergistic effects, both in vitro and in vivo. Compared with controls, the siRNA- and DOX-loaded MSNs induced 96.4% tumor growth inhibition in vivo, while preserving the bodyweight of treated mice. That formulation also reduced DOX accumulation in the liver and kidney and significantly increased accumulation in the tumor tissues due to the enhanced permeability and retention (EPR) effect. Among the major organs, nanoparticles showed higher accumulation in lungs due to their abundant blood supply. After cellular uptake, the high concentration of intracellular glutathione triggered the release of the payloads [48]. 

On the other hand, to enhance the uptake of the nanosystems in targeted cells, active targeting strategies have also been employed, using targeting ligands, such as folic acid, antibodies, or lactobionic acid, which have a specific affinity to the receptors overexpressed on the surface of cancer cells [11,20,21]. Zhang and colleagues, for instance, designed a nanocarrier based on the active targeting of asialoglycoprotein receptor (ASGPR) using amino-modified MSNs conjugated with lactobionic acid (LA), for the co-delivery of sorafenib and vascular endothelial growth factor (VEGF)-targeted siRNAs (siVEGF) to hepatocellular carcinoma (HCC) [49]. In this study, the nanocarrier was approximately spherical with an average diameter of 148.5 nm and it was able to enhance the anti-cancer efficacy of sorafenib and siVEGF and significantly inhibited the expression of angiogenesis-related VEGF proteins in Huh7 cells [49].

In a recent work by Choi and colleagues, a non-conventional and amine-free surface modification of MSNs was described, employing calcium ions (Ca^2+^) as a cationic “glue” between the negatively charged surface silanol groups of the nanoparticles and the phosphate backbone of the genetic material [50]. The toxicity of the developed nanocarrier was evaluated in vitro (cytotoxicity) and in vivo (immunogenicity) and compared to that of APTES- or PEI-coated MSNs, which are commonly reported in literature. Interestingly, the Ca^2+^-coated MSNs revealed to be highly biocompatible, much less cytotoxic and with no noticeable immunogenic response, when compared with the amine-coated MSNs, presenting similar results as the unmodified MSNs. Moreover, the therapeutic potential of these nanocarriers was assessed in vivo by the simultaneous delivery of a pore-loaded drug (DOX) and surface-loaded siRNA targeting the anti-apoptotic Bcl-2 gene to a mouse model of ovarian cancer. A tumor suppression rate of ~72% was achieved compared to the bare MSNs control, which illustrated the effective transfection and apoptotic action of DOX and Bcl-2 silencing [50].

Despite having presented some successful results, in the studies described above, the genetic material was found to be mostly adsorbed on the surface of the nanoparticles instead of being loaded inside the pores, due to the limited pore size of the MSNs. This location of genes on the outer surface makes them less protected and more prone to enzymatic degradation, resulting in less successful transfection [11]. Therefore, MSNs with larger pore sizes, of up to 30 nm, have been synthesized using swelling agents [51] or even different hydrothermal treatments [52,53], in order to enhance the internal storage of the bio-macromolecules and thus their protection (Table 2).

**Figure 9:**
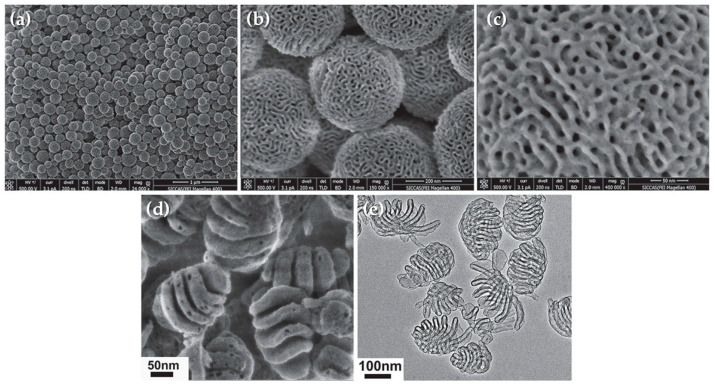
Mesoporous silica nanospheres with tunable pore structure. SEM images of large-pore mesoporous silica nanoparticles in different magnifications: (**a**) 24,000×; (**b**) 150,000× and (**c**) 400, 000×; and flower-like mesoporous nanoparticles obtained with less surfactant: (**d**) SEM image; (**e**) TEM image. Adapted from [55], with permission from John Wiley and Sons.

By simply incorporating the swelling agent 1,3,5-trimethlybenzene (TMB), Kim and colleagues synthesized MSNs of about 250 nm with ultra-large pores (~23 nm) [51]. The resulting MSNs were surface modified by post-grafting with APTES and their performance compared with MSNs with small pores (~2 nm). The MSNs with large pores showed efficient cellular uptake and higher capacity to load plasmid DNA than those with small pores, where the zeta potential results suggested the presence of pDNA at the surface of the particle. Moreover, MSNs with large pores demonstrated a remarkable ability to protect plasmids from nuclease degradation and much higher transfection efficiency than MSNs with small pores [51]. Another example of MSNs with large pores was developed using ethanol as a co-solvent and fluorocarbon-hydrocarbon mixed surfactants as templates [54]. In this work, particles with 9 nm pores were obtained and further modified with hydrophobic octadecyl groups. The resulting systems showed efficient loading capacity and a delivery efficiency of functional siRNAs to human colon cancer cells (HCT-116) comparable to a commercially available transfection reagent (Oligofectamine^TM^), leading to inhibition of cancer cell growth [54]. Dong’s group has also developed MSNs with large pores of 20 nm using a mixture of both these methods (TMB as a swelling agent and a dual surfactant system) and a low temperature (10 °C) synthesis [52]. The final properties of the MSNs were achieved through a subsequent hydrothermal treatment (100–150 °C) and further surface modification with aminopropyl groups. The developed nanoparticles were able to encapsulate a noticeably high content of DNA inside the pores and protect it from enzymatic degradation [52]. A different research group reported the development of monodispersed MSNs with ordered and interconnected large pore channels which, interestingly, could be tuned from hexagonal to cubic or lamellar mesostructures (Figure 9) by simply controlling the amount of surfactant [55]. Moreover, by varying the length of the poly(acrylic acid) (PAA) used as template, the morphology of the nanoparticles could be adjusted from large-pore to hollow mesoporous silica nanoparticles. In vitro and in vivo tests were performed with nanoparticles after amino groups modification, and they revealed high pDNA loading and transfection efficiency into SMMC-7721 cells, showing no significant differences between their performance and that of commercially available Lipofectamine^®^ 2000. Moreover, after a 21-day treatment of subcutaneous injections of the pDNA-loaded nanoparticles to tumor-bearing mice every 3 days, tumor volume was effectively reduced by 47% in treated mice, as compared to the control groups without treatment, treated with free-pDNA and with the empty nanoparticles, due to the demonstrated VEGF gene silencing and inhibited angiogenesis [55].

A different pore enlargement strategy has also been reported by Shi’s group [53], by taking advantage of the differences between the chemical-bond stability within organosilica nanoparticles’ framework, which will be further detailed in the section below.

#### Organosilica Nanoparticles

Organic groups have been successfully incorporated into the framework of silica nanoparticles by mixing different bissilylated precursors with the traditionally used TEOS. Opposite to the chemically inert and slowly hydrolyzed pure silica nanoparticles, different organic groups in the framework of organosilica nanoparticles can slower or accelerate the degradation behavior of the nanoparticles and confer them tumor microenvironment (pH, redox, or enzyme)-triggered biodegradation properties, which can be used for the controlled release of loaded cargos [19,56]. For instance, by inserting cleavable sulfide bonds within the silica framework, redox-responsive nanoparticles, able to benefit from the significantly higher glutathione (GSH) concentration within cancer cells, have been developed for several biomedical applications (recently reviewed in [19]). 

**Figure 10:**
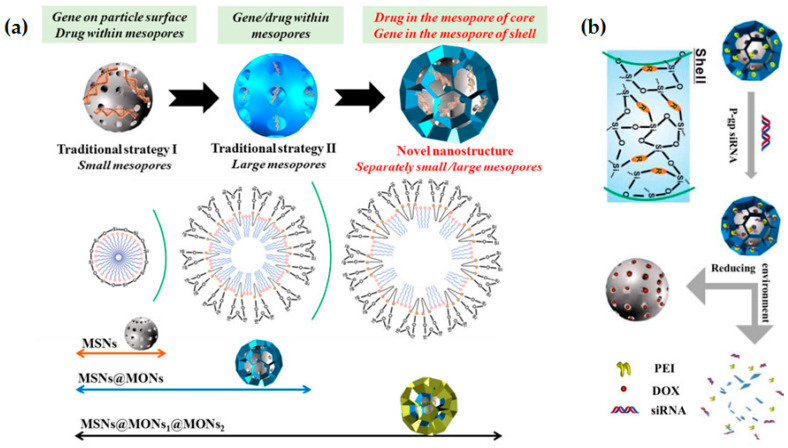
(**a**) Schematic illustration of the structural evolution and formation mechanism of MSNs from small to large pore-sized MSNs, and core-shell hierarchical MSNs (with small and large pores for drug and gene co-delivery); (**b**) Breaking-up of the mesoporous organosilica shell of the nanoparticles under reductive environment and consequent siRNA and DOX release. Adapted from [58], with permission from Elsevier.

As mentioned in the section above, the introduction of organic groups in the framework of silica nanoparticles also allows different approaches for pore-enlargement of MSNs. Shi’s group employed 1,4-bis(triethoxysilyl)benzene as an organosilica precursor to obtain hollow mesoporous organosilica nanoparticles with abundant Si-C bonds and performed a selective breakage of those bonds, which are significantly weaker than Si-O bonds, through hydrothermal treatments, resulting in ultra-large pores of about 24 nm [53]. The same research group had already reported the use of bis[3-(triethoxysilyl)propyl] tetrasulfide (BTESPT) to develop flower-like mesoporous organosilica nanoparticles (MONs) with large pores (6.2 nm) and small particle size (~30 nm), which were successfully employed for the delivery of pDNA after stepwise surface modifications with PEI, for genetic material binding, and the cell-penetrating peptide transactivator of transcription (TAT), for intra-nuclear gene delivery. The nanoparticles demonstrated enhanced loading capacity of pDNA and efficient protection from nuclease-degradation, as well as high intra-nuclear transfection efficiency in HeLa cells [57]. Later on, and once again using BTESPT, they developed a different and unique type of MSNs, which were designed containing both small and large mesopores, in order to facilitate the independent encapsulation of small (doxorubicin) and large (siRNA targeting P-glycoprotein (P-gp)) molecules, as illustrated in Figure 10a [58]. The developed nanoparticles presented a core-shell hierarchical mesoporous structure with small pores (~3 nm) on the core and larger pores (4–9 nm) on the shell layer. In addition, the framework of the organosilica shell contained tetra-sulfide bonds, which enable a controlled release of the siRNA in response to the reductive tumor microenvironment (Figure 10b). The successful performance of the system was proven both in vitro and in vivo: it showed enhanced transfection efficiency in MCF-7/ADR cells and suppression of the P-gp expression, as well as significant inhibition of tumor growth in vivo [58]. In the same way, silica nanoparticles with disulfide bond-bridged silica framework have also been reported for gene/drug co-delivery, using the commercially available silane bis[3-(triethoxysilyl)propyl] disulfide (BTESPD) [59], or even smartly designed ones, such as in the work performed by Zhang and colleagues, where the disulfide-bridged silane was fabricated by reacting 3-(triethoxysilyl)propyl isocyanate (IPTS) with cystamine dihydrochloride [60].

**Figure 11:**
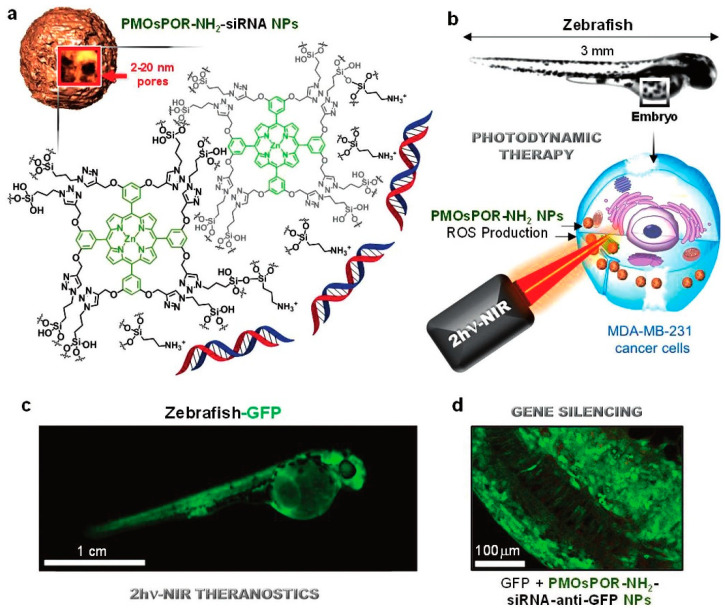
(**a**) Amine functionalized porphyrin-based mesoporous organosilica nanoparticles (PMOsPOR-NH_2_) for siRNA binding; (**b**) Two-photon excited near-infrared (TPE-NIR) photodynamic therapy on zebrafish via PMOsPOR-NH_2_; (**c**) TPE-NIR gene silencing on zebra fish-GFP via PMOsPOR-NH_2_-siRNA; (**d**) Bioimaging of zebrafish embryos after gene silencing. Reproduced from [61], with permission from John Wiley and Sons.

More recently, much larger stimuli-responsive precursors have been used in the development of organosilica nanoparticles for gene delivery applications. In a work by Jimenez and colleagues, porphyrin-based organosilica nanoparticles were developed for theranostics combined with gene delivery [61]. The nanoparticles of 100–300 nm in diameter presented interconnected large cavities of 10–80 nm and their framework composed of J-aggregates of porphyrins allowed near-infrared two-photon excitation of the nanosystem for imaging and photodynamic therapy (PDT) consisting of the production of reactive oxygen species and consequent disruption of the endosomal membranes and release of cargos (photochemical internalization) and cell killing. The organosilica nanoparticles were also functionalized with APTES for siRNA binding, and the aminated nanoparticles showed to be very efficient for anti-cancer PDT in MCF-7 and MDA-MB-231 cancer cells and even in vivo in a zebrafish model (Figure 11). Moreover, when complexed with siRNAs targeting the luciferase gene and incubated with luciferase-expressing MCF-7 cancer cells, 50% of gene silencing was achieved after PDT. Anti-GFP siRNAs were also successfully delivered in a zebrafish model [61]. Building upon this study, and based on the fact that phthalocyanines may be more appropriate for PDT than porphyrins, since they present better NIR-light absorption properties, phthalocyanine-based organosilica nanoparticles have also been reported for photodynamic therapy and photochemical internalization-mediated gene delivery. In this study, 50-nm nanoparticles were obtained and amination with APTES was also performed. Here, incubation of the siRNA-containing nanosystems with MCF-7 cells resulted in 64% of luminescence decrease after near-infrared light irradiation, showing the efficiency of photochemical internalization [62].

## 5. Hybrid Silica Nanoparticles

### 5.1. Polymer Modified Silica Nanoparticles

Among the large number of materials that have been used to functionalize the surface of silica nanoparticles, polymers have drawn increasing interest due to their potential to address biomedical applications and their exceptional chemical and structural versatility. The vast availability of monomers, the ease of synthesis, and the possibility to control parameters such as the molecular weight of the polymer, allows the design of countless different polymeric structures with different architectures and properties. Therefore, it is possible to create tailor-made polymers that attend the desired characteristics, such as hydrophilicity or hydrophobicity, surface charge or even responsiveness to certain stimuli [63,64].

In the last decade, different cationic polymers, such as polyethylenimine (PEI) [65,66,67,68,69,70,71,72,73], poly(2-(dimethylamino)ethyl methacrylate) (PDMAEMA) [74,75], chitosan derivatives [76], poly l-arginine [77] or poly l-lysine (PLL) [78], have been reported as coatings for silica nanoparticles in gene delivery systems, either through simple electrostatic interactions with the surface silanol groups, through covalent silane coupling with trialkoxysilanes, or by amidation reactions of amine-containing polymers with carboxylated NPs [20]. Table 3 summarizes a variety of different polymer-modified silica-based gene delivery formulations recently reported in literature.

Among the existing cationic polymers, PEI is the most commonly used and is generally accepted as the gold standard for nonviral gene delivery [15,64]. This polymer has a high density of amine groups which, not only enhance the electrostatic interaction with genetic material and the cellular uptake but also help to avoid the endo/lysosomal pathway degradation due to the “proton sponge effect” [64,69]. This process is characterized by the protonation of amine groups upon acidification in the endosome, leading to increased osmotic pressure and eventual swelling and burst of the endosome, consequently releasing the nanosystem into the cytoplasm [79,80]. Despite their advantages and efficiency to transfect cells, PEI and other high molecular weight polycations, can present significant cytotoxicity namely by perturbing plasma membranes. Studies comparing the performance of nanosystems created with PEI of different molecular weights have been showing that 25 kDa PEI results in high transfection efficiency but has also severe cytotoxicity. On the other hand, reducing the molecular weight efficiently reduces the cytotoxicity but can also significantly affect the transfection efficiency [20]. Several studies have reported MSNs coated with 10 kDa PEI as a suitable carrier for gene delivery, presenting better overall results than the same nanoparticles coated with 25 kDa PEI, 1.8 kDa PEI or the polymers alone [21,65,67,68].

Polyethylene glycol (PEG), which is a neutral, highly hydrophilic and biocompatible polymer, has also been used as a steric layer in polycation-coated MSNs, in order to help minimize the system cytotoxicity and shield it from RES recognition [65,73]. PEGylation is the most commonly used strategy to minimize in vivo opsonization and clearance of silica nanoparticles, enhancing their stability and circulation lifetime [85,86]. Nonetheless, the use of PEG concurrently compromises the cellular uptake, which is sometimes balanced by the use of a ligand-mediated active targeting strategy [20]. To that end, Ngamcherdtrakul and colleagues used MSNs coated with a PEI-*b*-PEG copolymer to deliver siRNA to HER2+ breast cancer cells [65]. While PEI was crosslinked to enhance its buffering capacity and endosomal escape, PEG coating protected siRNA from degradation and prevented nanoparticle aggregation, enhancing the safety and efficiency of the system. Furthermore, a monoclonal antibody (trastuzumab) was used as a ligand for the selective uptake of the nanoparticles by the HER2-overexpressing cancer cells. The nanosystem effectively silenced in vitro HER2 expression without causing a significant adverse immune response and was also able to significantly inhibit in vivo tumor growth [65]. A similar nanosystem was also used for the targeted delivery of siRNA against Polo-like kinase 1 (PLK1) in a triple-negative breast cancer (TNBC) model [87]. PLK1 is considered a proto-oncogene involved in cell division commonly overexpressed in tumor cells and has been identified as the strongest therapeutic target for TNBC cells. In this study, the nano-construct reduced tumor incidence in the lungs and other organs in mice model, and long-term treatment significantly delayed the onset of death and improved the overall survival of TNBC mice. Additionally, it was shown that the nanosystem itself (unloaded) was able to scavenge intracellular ROS and modulate NOX4 activity, which in turn inhibited cancer cell migration and invasion in TNBC cells in vitro [87].

Using disulfide linkers, Rosenholm’s group has also developed PEI-coated MSNs for redox-responsive intracellular siRNA delivery [71,72]. The MSNs were functionalized with covalently attached hyperbranched PEI and redox-cleavable linkers that were able to carry a high payload of siRNA (120 mg/g). After siRNA loading, a second capping layer of low molecular weight PEI (1.3 kDa) was attached to the exterior surface in order to improve the stability of the encapsulated nucleic acids and to enhance the cellular uptake and endosomal escape of the nanosystem. The authors have shown the nanoparticles to be not toxic for MDA-MB 231 cells and that the transfection efficiency was comparable to that obtain with the commercial transfection agent Lipofectamine [71,72].

Bhattarai and colleagues tested PDMAEMA and PDEAEMA polymers conjugated with PEG as coatings for MSNs for gene delivery and found that all the nanosystems (MSNs-PEG, MSNs-PEG-PDMAEMA and MSNs-PEG-PDEAEMA) were non-cytotoxic to murine melanoma cells even though MSNs coated with PDMAEMA revealed the best pDNA transfection efficiency [74]. In fact, those nanocarriers were able to successfully complex both pDNA and siRNA, and transfect in vitro, resulting in 50% decrease of luciferase expression and 20% GADPH suppression, when delivering anti-luciferase and anti-GADPH siRNAs, respectively. The nanoparticles were also loaded with a lysosomotropic agent—chloroquine (CQ)—which further enhanced the transfection activity with both plasmid DNA and siRNA, demonstrating the suitability of the system for drug and gene co-delivery [74]. Also using PDMAEMA as a polymeric coating for silica nanoparticles, Lin and co-workers studied the effect of particle size and shape on transfection and showed that the morphology of the nanoparticles plays an important role in the transgene expression, especially when the polycation amount is low, a larger aspect ratio being favored [75]. Chiral nanorods 300 nm-long-based systems were found to be the most efficient gene carriers. They also found that hollow nanoparticles-based carriers had better transfection performance than solid counterparts and almost all the polymer-coated nanohybrids presented higher transfection efficiencies than control PEI (25 kDa) or PDMAEMA [75].

**Figure 12:**
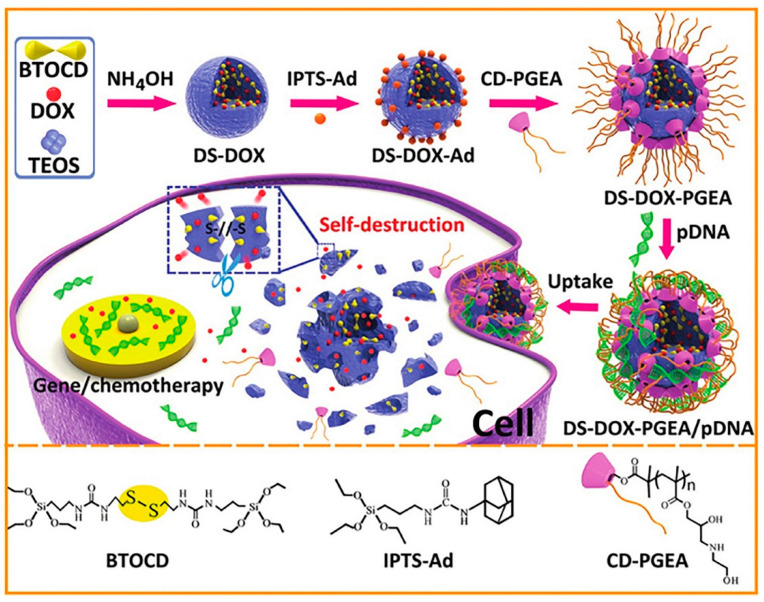
Schematic illustration of the redox-responsive drug/gene co-delivery system. DOX was embedded in a disulfide-silica network, while pDNA was complexed with the polymeric coating, which promoted the self-destruction of the nanosystem and cargo release upon cellular internalization. Adapted from [60], with permission from John Wiley and Sons.

A drug and gene co-delivery system was developed by Zhang and colleagues combining disulfide-bridged and doxorubicin (DOX)-embedded silica nanoparticles with a polycation coating comprising one β-cyclodextrin core and two ethanolamine-functionalized poly(glycidyl methacrylate) arms (Figure 12) [60]. The embedded DOX facilitated the degradation of the silica nanoparticles framework due to the weak silica condensation which, interestingly, resulted in enhanced release and simultaneous delivery of DOX and pDNA encoding p53 after cellular uptake. This co-delivery presented synergistic effects, not only in vitro but also in C6 glioma tumor-bearing mice [60]. Wang’s group also developed a redox-responsive co-delivery system for DOX and p53 gene [76]. Nonetheless, in this work, a chitosan derivative—poly(amidoamine) (PAMAM) dendrimer (G2) conjugated to chitosan—was grafted on the MSNs’ surface via a disulfide linker, acting simultaneously as a “gatekeeper” for the controlled release of DOX and as a complexation and protection site for pDNA. After cellular internalization (under a glutathione-rich environment), the polymer shell was shed, leading to the accelerated release of DOX and nucleic acids. The developed nanosystem presented good biocompatibility and high gene transfection efficiency. The co-delivery of p53 DNA and DOX also revealed synergistic effects and induced significant apoptosis in HeLa cells [76]. Also different synthetic and pH-responsive polymers, such as poly(β-amino ester) [53] or poly(glycidyl methacrylate)-*b*-poly(2-(dimethylamino)ethyl methacrylate) (PGMA-*b*-PDMAEMA) [88] have recently been reported as successful choices in the development of MSN-based nanosystems for co-delivery of chemotherapeutic drugs and nucleic acids.

**Figure 13:**
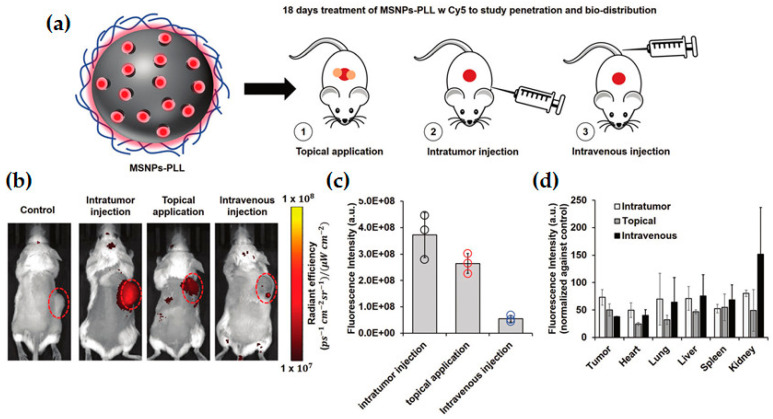
Biodistribution studies of Cy5-marked PLL-coated MSNs: (**a**) Illustration of the administration routes tested for in vivo studies in squamous cell carcinoma (SCC) mice xenograft model; (**b**) Representative IVIS images of mice treated with Cy5-marked PLL-MSNs; (**c**) Fluorescence quantification of IVIS images; (**d**) Intensity of fluorescence of isolated tumor and major organs after 18-day treatment. Adapted from [78], with permission from Royal Society of Chemistry.

Polypeptides, composed of naturally occurring amino acids, have also been used as coatings for MSNs due to their higher biocompatibility and less toxicity when compared to most synthetic cationic polymers such as PEI [11,89]. Kar and colleagues synthesized poly l-arginine-grafted MSNs for DOX and pDNA co-delivery, and obtained not only great complexation of DNA with peptide, but also excellent cell uptake in both HeLa and A549 cells (>90%) [77]. The nanocarriers proved to be almost non-cytotoxic (85% cell viability even at 100 μg/mL) and reached up to 60% transfection efficiency [77]. PLL polymers have also been commonly used for gene delivery purposes due to their low immunogenicity and great DNA loading ability [11,90]. Lio and co-workers recently developed a topical formulation based on PLL-coated MSNs for the transdermal delivery of siRNA [78]. In vivo biodistribution studies in tumor-bearing mice compared the topical application with intratumor and intravenous administration of the Cy5-labeled nanosystem, as presented in Figure 13a. Both intratumor injection and topical application resulted in a localized distribution of Cy5 in mice tumor, with similar fluorescence intensity, which was much higher than that observed after intravenous injection, as revealed by IVIS imaging results (Figure 13b,c). Furthermore, transdermal delivery revealed a targeted delivery into the tumor, with excellent penetration of Cy5 in the skin and tumor, and minimal accumulation of nanoparticles in the vital organs, opposite to the injection administration methods (Figure 13d), minimizing toxicity concerns. Additionally, the system enabled the topical delivery of siRNA targeting transforming growth factor beta receptor 1 (TGFβR-1), which is overexpressed in about 80% of patients with squamous cell carcinoma (SCC) of the skin, its suppression presenting anti-tumor effects. An 18-day topical treatment in a mouse tumor xenograft model demonstrated the successful gene regulation effect of the system, resulting in a 2-fold abolishment of TGFβR-1 expression in tumor tissue and approximately 2.5-fold tumor growth inhibition, in comparison to scrambled siRNA or the PBS-treated control [78]. Möller’s work describes a different gene delivery system that comprises artificial amino acids to form a modularly designed block copolymer, in combination with oleic acid blocks, to act as a capping and endosomal release agent of MSNs [81]. The nanocarrier demonstrated to be non-toxic and presented highly effective cell transfection and siRNA delivery into KB-cells, revealing up to 80–90% luciferase knock-down even at extremely low cell exposures [81].

Contrary to the polycationic-coating strategies described above, MSNs coated with hyaluronic acid (HA), a natural occurring anionic polysaccharide, have also been reported for use in gene delivery [11]. Several studies have focused on the application of HA as a specific ligand for active targeting of CD44-overexpressing cells, such as the human lung cancer cell line A549 [11,21,59,91]. Therefore, the HA coating of MSNs for gene delivery purposes, serves not only as a protection for nucleic acids but also as a targeting agent to cancer cells. Li and colleagues developed HA-siRNA conjugates via disulfide links, which were electrostatically complexed with amine-modified silica nanoparticles [82]. The resulting complexes were efficiently internalized in A549 cells and significantly inhibited GFP expression without severely affecting cell viability, as compared to the control PEI (25 kDa). The targeting ability of the HA-coated nanocarriers was also confirmed by an in vivo biodistribution assay in A549 tumor-bearing mice model [82]. Furthermore, Shi and co-workers have recently reported the successful delivery of TH287 in oral squamous cell carcinoma using HA-coated MSNs [83]. TH287 is a small drug able to inhibit the protein MutT homolog 1 (MTH1), which is known to hydrolyze the oxidized forms of dATP and dGTP, preventing their misincorporation in DNA and thus inhibiting cancer cell proliferation. Therefore, in a situation of increased oxidative stress, down-regulation of MTH1 expression can reduce tumor cell survival and growth. Simultaneously, the nanosystem was used to deliver siRNA targeting MDR1, also known as P-gp, which is a cell membrane protein responsible for the drug resistance of tumor cells (by pumping out anticancer drugs and preventing their intracellular accumulation). Cytotoxicity and apoptosis assays showed the synergistic effect of drug and gene co-delivery compared to that of TH287 alone. The use of HA-coated nanosystems led to their effective internalization by CAL27 cells and their antitumor efficacy was tested in vivo revealing a 4-fold decrease in tumor growth compared to that of control and 2-fold compared to that of non-coated MSNs. The results showed the dual effect of the system in inhibiting the MDR1 function and enhancing the cell-killing effect of the TH287 drug in tumor tissues [83].

Li and colleagues recently reported the use of a polymerized dopamine (PDA) film as a pH-responsive gatekeeper for anti-miR-155-loaded MSNs, which were also further modified with an SH-terminated AS1411 aptamer for the targeted treatment of colorectal cancer (CRC) [84]. mir-155 is one of the most important oncogenes, regulating several cancer-related pathways as well as drug resistance and genome instability [92]. In this work, in vitro and in vivo data revealed that the nanosystem was able to efficiently target CRC tumor, due to both active targeting of AS1411 aptamer and passive targeting by EPR effect, and downregulate miR-155 expression, resulting in a significant tumor growth inhibition. Moreover, it was shown that treatment with this nanosystem could resensitize CRC tumor to the chemotherapeutic drug 5-fluorouracil by downregulating P-gp [84].

### 5.2. Lipid Modified Silica Nanoparticles

Lipids are a class of biomacromolecules characterized by their hydrophobic or amphiphilic nature, which includes fats, sterols, phospholipids, glycerides, or fat-soluble vitamins. Their ability to form vesicles and membranes, as well as their biocompatibility and non-immunogenicity, makes them attractive candidates for the development of nanocarriers, with liposomes being one of the most greatly investigated delivery system, not only for nucleic acids but also for small molecule drugs [7,21,93]. Thus, surface engineering of inorganic nanoparticles, such as silica nanoparticles, with a lipid shell is a very well-accepted method for the production of hybrid nanosystems with synergistically combined characteristics [5,21]. Additionally, the surface of lipid-coated nanoparticles can be easily modified for the addition of targeting ligands, PEGylation, or other functional moieties, by simultaneous or postinsertion of lipid-conjugates [5]. For instance, van Schooneveld and colleagues investigated the effect of a physically adsorbed monolayer of PEGylated phospholipids on silica nanoparticles’ in vitro and in vivo performance, namely short-term cytotoxicity and pharmacokinetics, and reported that the lipidic coating improved the applicability, biocompatibility, and pharmacokinetics of these nanoparticles. The PEG-lipid shell prevented silica nanoparticles from aggregating, increased blood circulation half-life time by 10-fold (165 min vs. 15 min) and resulted in a more favorable tissue distribution profile [94]. It was observed that lipid-coated nanoparticles accumulated gradually in the liver and spleen, while bare nanoparticles accumulated much faster and in a higher extent, highlighting their possible opsonins exposure and thus poorer pharmacokinetics. Additionally, bare nanoparticles accumulated in the lungs, causing breathing problems and liver necrosis, while none of that or other adverse effects were observed with coated nanoparticles [94].

Due to their attractive properties and ability to encapsulate a variety of different cargos, in the last decade lipid-encapsulated MSNs have drawn the attention of the scientific community and have been reported as nanocarriers for a range of different therapeutic strategies. For instance, as drug delivery systems [95,96,97,98,99,100] or for protein delivery [101], but also as vectors for combined therapies, such as gene and drug co-delivery [102] or co-delivery of different therapeutic nucleic acids [103].

**Figure 14:**
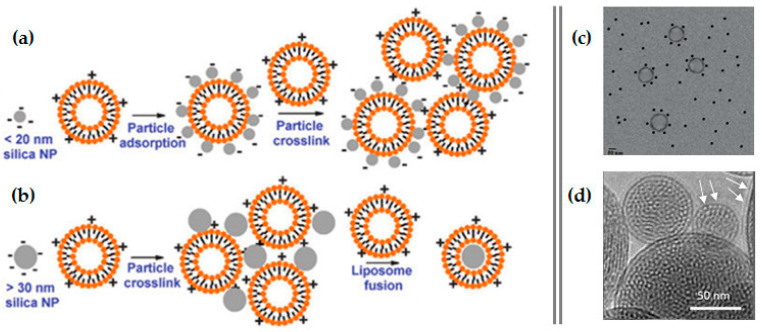
Illustrative representation of the interaction between silica nanoparticles and cationic liposomes (**a**,**b**), and TEM images (**c**,**d**) of the respective results: (**a**) Small silica NPs are adsorbed onto the liposomes, resulting in a negatively charged surface. (**b**) Larger silica NPs lead to the formation of supported bilayers with a positively charged surface, which are useful for DNA binding. (**c**) TEM image of 10–20 nm silica NPs prepared with 1,2-dimyristoyl-sn-glycero-3-phosphocholine (DMPC) small unilamellar vesicles. (**d**) Cryogenic TEM image of the protocell, showing the mesoporous silica nanoparticle core and the SLB (white arrows). ((**a**,**b**) adapted from [104] with permission from Royal Society of Chemistry; (**c**) Adapted from [107] with permission from American Chemical Society; (**d**) adapted from [108] with permission from Elsevier).

**Figure 15:**
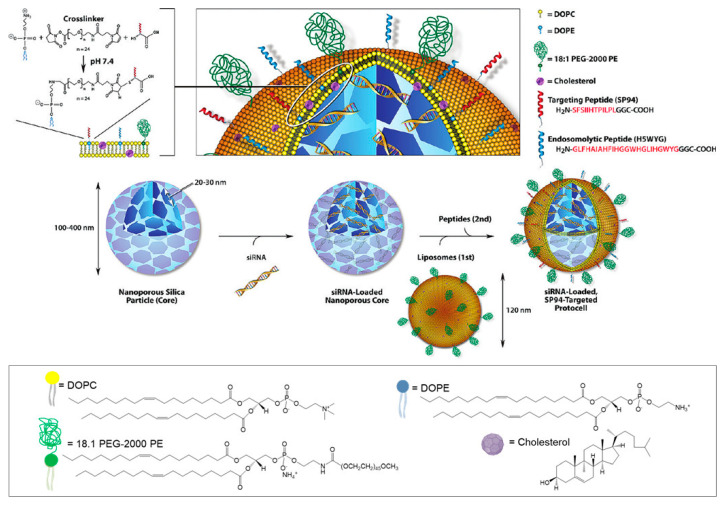
Schematic illustration of the synthesis of siRNA-loaded mesoporous silica nanoparticle-supported lipid bilayers (protocells). Reproduced from [106], with permission from American Chemical Society.

On the matter of developing lipid-coated MSNs for gene delivery, Brinker’s group was a pioneer [104,105,106]. They tested the interaction of silica NPs with cationic liposomes and DNA and found that if the NPs are too small (<20 nm), they end up adsorbed onto the positively charged liposomes, which form very large aggregates, hindering DNA binding (Figure 14a,c) [107]. On the other hand, larger silica NPs (30–130 nm) resulted in the formation of NP-supported lipid bilayers (SLB), with positive surface charges, and thus able to successfully bind the negatively charged DNA (Figure 14b) [104]. Moreover, by fusing liposomes to high-surface-area MSNs and further modifying the resulting SLBs with a targeting peptide, a fusogenic peptide, and PEG, this research group developed a new nanocarrier construct referred to as “protocell” (Figure 14d). Compared to the traditional liposomes, this nanosystem has demonstrated greater bio-applicability, since it is able to encapsulate a variety of different cargos [105]. Ashley and colleagues developed amine-functionalized MSNs with large pores (23–30 nm) that were able to load about 1 nmol of siRNA per 10^10^ particles [106]. After coating with a cationic (1,2-dioleoyl-3-trimethylammonium-propane (DOTAP))- or zwitterionic (1,2-dioleoyl-sn-glycero-3-phosphocholine (DOPC))-based lipid shell, the loading ability of the nanosystems was not impaired. On the contrary, the resulting SLB had a loading capacity 10–100 times higher than that of the corresponding liposomes. Furthermore, a targeting peptide that binds to HCC (SP94) and an endosomolytic peptide (H5WYG) that promotes endosomal/lysosomal escape were added to the surface of these protocells, as illustrated in Figure 15. In vitro studies have revealed that the nanosystem loaded with an equimolar mixture of siRNAs targeting cyclin A2, B1, D1 and E was able to silence each of the target proteins expression by nearly 90% in 72 h, presenting remarkably low IC90 values for Hep3B cells with almost no adverse effects on normal hepatocytes [106]. From the same research group, Dengler and co-workers reported the use of a very similar protocell construct to deliver pDNA encoding the anti-inflammatory and pain-suppressive therapeutic transgene, interleukin-10 (pDNA-IL-10), to the spinal cord. In this study, due to the large size of the pDNA cargo, the positively charged MSNs with small pore size (2–5 nm) did not load the genetic material inside the pores, instead it was adsorbed onto the surface of the MSNs and it was hypothesized that it was trapped between the MSN and the lipid layer. Intrathecal (i.t.) administration of both DOTAP-cholesterol-based and DOPC-based protocells was well-tolerated in peri-spinal region of rats and the animals experienced no adverse effects, even at the highest dose of protocells. Moreover, i.t. injection of pDNA-IL-10 loaded on DOTAP-Chol protocells was able to effectively reverse pain thresholds for almost 2 weeks [108].

In a different study, liposome-silica hybrid nanoparticles were also used for the delivery of a synthetic double-stranded RNA (dsRNA) analog, polyinosinic-polycytidylic acid (poly(I:C)), able to modify cancer microenvironment and suppress tumor growth by both upregulating tumor suppressor genes and activating cell apoptosis via toll-like receptor 3 (TLR3), retinoic acid inducible gene I (RIG-I) and melanoma differentiation-associated gene-5 (MDA5). For this work, the lipid shell covering the MSNs was formed by a four-component lipid system using two cationic lipids, DOTAP and (3β-[*N*-(*N*′,*N*′-dimethylaminoethane)-carbamoyl])-cholesterol (DC-Chol), and two zwitterionic lipids, DOPC and dioleoylphosphatidylethanolamine (DOPE), and PEGylated and non-PEGylated nanosystems were tested in prostate PC3 and breast MCF7 cancer cell lines. It was notably found, that negatively charged poly(I:C)-loaded hybrid nanoparticles were able to efficiently kill cancer cells even at extremely low poly(I:C) concentrations, being much more effective than their liposome counterparts, which required about 10-fold more poly(I:C) to induce a similar apoptosis level [109].

**Figure 16:**
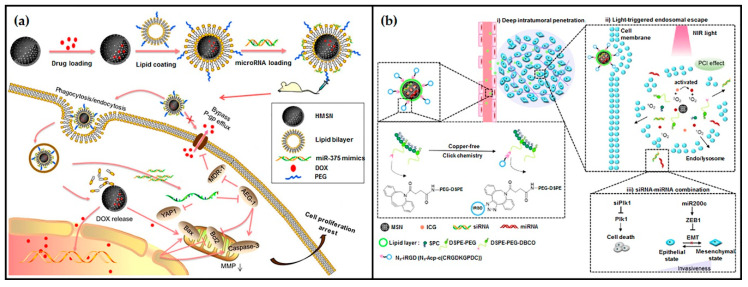
Schematic illustrations of recently reported combination therapies using lipid-coated silica nanoparticles: (**a**) Development and antitumor mechanism of LHD/miR-375 (reproduced from [102], with permission from Dove Medical Press Limited); (**b**) iRGD-modified lipid-coated MSNs and light-triggered siPlk1/miR-200c delivery mechanism (reproduced with permission from [103], copyright 2020, American Chemical Society).

To attain the synergistic therapeutic effects of gene and drug co-delivery, Xue and co-workers developed PEGylated lipid-coated hollow MSNs enclosing DOX and loaded with miR-375 (called LHD/miR-375), as presented in Figure 16a, for in vitro and in vivo delivery to multidrug-resistant HCC cells [102]. The nanosystem was able to suppress the expression of P-gp in HepG2/ADR cells, resulting in a significant decrease in the DOX efflux and thus in an enhanced antitumor effect. This synergistic effect between DOX and miR-375 was greatly illustrated in the much lower IC_50_ values of LHD/miR-375 in all the tested cell lines, when compared to that of free-DOX or encapsulated DOX only. Additionally, LHD/miR-375 could also significantly enhance the DOX antitumor effects and suppress tumor growth in tumor-bearing mice, while presenting a much better safety profile than DOX [102]. Wang and colleagues, on the other hand, recently reported the use of lipid-stabilized MSNs for simultaneous delivery of siRNA against PlK1 and mir-200 c. In this work, besides PEGylation, the lipidic shell was conjugated to the iRGD peptide to facilitate deep tumor penetration, and indocyanine green (ICG), a near-infrared-responsive photosensitizer, was encased into the MSNs to enhance endosomal escape through the light-activated generation of reactive oxygen species (ROS) (Figure 16b). An increased cellular uptake of the nanosystem was observed in both in vitro 3D tumor spheroids and in vivo orthotopic MDA-MB-231 breast tumors. In addition, the systemic administration of the nanoparticles in the orthotopic breast cancer model resulted in a significant suppression of the primary tumor growth and in considerable antimetastatic activity upon short light irradiation, which are promising results for future metastatic cancer treatment [103].

### 5.3. Magnetic Silica Nanoparticles

In the last decades, magnetic nanoparticles such as iron oxides have been extensively studied in different fields. In particular, iron oxide modified silica nanoparticles have been used as contrasting agents for magnetic resonance imaging (MRI), showing remarkable enhancement of the MR signal [110,111,112,113]. The use of silica coatings has revealed an improvement in the chemical stability of the magnetic nanoparticles and reduction of their toxicity, so conjugation of mesoporous silica nanoparticles with magnetic nanoparticles has been arousing great interest in nanomedicine as targeting tools for theranostics [3]. Their combined properties allow the co-delivery of therapeutic and imaging functions with the convenience of magnetically guided delivery (magnetofection), as the systems can be led to target sites by controlling the external magnetic field (EMF), increasing the therapeutic efficiency and minimizing the non-specific distribution [114,115,116].

Numerous studies on the combination of magnetic nanoparticles with mesoporous silica have been reported, one of the methods often explored being the synthesis of large-pore sized MSNs and the impregnation of iron oxide within their pores in a later stage [55,115]. The main disadvantage of this method is the loss of cargo capacity since part of the pores space is occupied by the magnetic nanoparticles. For instance, Yu’s group developed large pore MSNs loaded with iron oxide by impregnation and surface modified with polyethyleneimine (denoted PEI-Fe-LPMSN) [115]. The system was used to deliver siRNA into osteosarcoma cancer cells and resulted in significantly higher cytotoxicity (80%) than the same dose of siRNA delivered by a commercial transfection reagent, Oligofectamine^TM^ (50%). Additionally, the application of a magnetic field caused an increase in cellular uptake efficiency [115].

**Figure 17:**
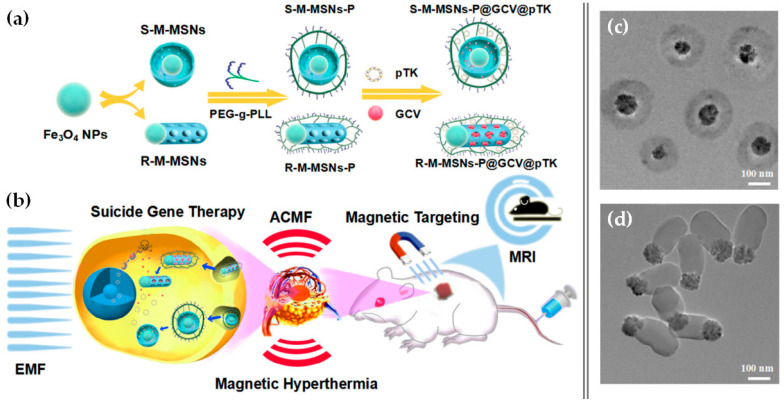
Schematic illustration of (**a**) the preparation of differently shaped magnetic-MSNs with a polymer (PEG-*g*-PLL) coating for herpes simplex virus thymidine kinase/ganciclovir (HSV-TK/GCV) gene therapy, (**b**) application of the nanosystem for MRI-guided, magnetically targeted and hyperthermia-enhanced suicide gene therapy of hepatocellular carcinoma. TEM images of the developed (**c**) sphere-like and (**d**) rod-like M-MSNs. Adapted from [116], with permission from Elsevier.

Another approach, and probably the most commonly used to fabricate magnetic mesoporous silica, is the development of core-shell nanoparticles with a magnetic core previously synthesized and a mesoporous silica shell condensed around it [117,118]. One drawback of this method is that the silica shell can lead to significant decrease in the saturation magnetization of the final nanoparticles, therefore, Xiong and colleagues developed nanoparticles with a super-paramagnetic magnetite cluster core and a radial large-pore mesoporous silica shell, which ensure a higher magnetization value of the final system than other types of Fe_3_O_4_/SiO_2_ nanocomposites [118]. The nanoparticles’ shape has also proven to have a great impact in their final properties, so within this type of structure, different designs and shapes have been studied, such as sphere-like [116,118,119], rod-like, sandwich-structured or rattle-type hollow magnetic mesoporous silica nanoparticles (M-MSNs) [116,117]. In a recent study, Wang and colleagues compared the performance of two differently shaped core-shell type M-MSNs in combined magnetically mediated suicide gene therapy and magnetic hyperthermia therapy for hepatocellular carcinoma (HCC) [116]. Magnetic hyperthermia is a promising noninvasive treatment for HCC patients, based on the capacity of magnetic nanoparticles to generate heat under an alternating current magnetic field (ACMF). In this study, carboxyl-functionalized MSNs with Fe_3_O_4_ cores were loaded with a drug (ganciclovir) and polyethylene glycol-grafted-poly(l-lysine) (PEG-*g*-PLL) was attached to the surface to allow the complexation of pDNA encoding the HSV-tk (Figure 17). They found that, compared with sphere-like MSNs, rod-like MSNs presented higher drug and plasmid loading capacity and better magnetic hyperthermia properties. The application of an EMF significantly enhanced the cellular uptake of pDNA and accumulation at the tumor site of both systems; however, a remarkable increase was observed with rod-like M-MSNs, revealing their stronger magnetically enhanced gene delivery. Mice receiving the loaded nanosystems plus the combined effect of EMF and an ACMF treatment had an astonishing tumor growth inhibition, which was smaller in the absence of either the EMF of ACMF [116].

**Figure 18:**
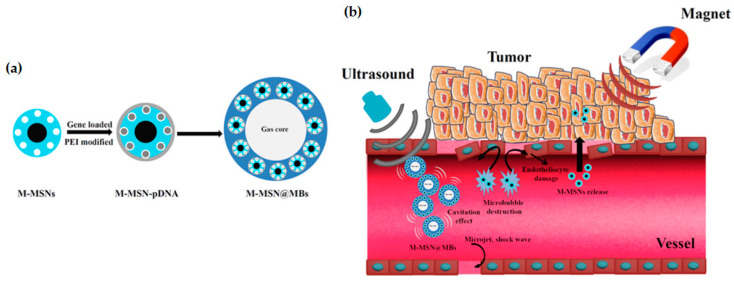
Schematic representation of: (**a**) construction of ultrasound responsive magnetic mesoporous silica nanoparticle-loaded microbubbles (M-MSN@MBs); (**b**) tumor-targeting gene delivery by M-MSN@MBs combining ultrasound and magnetic attraction. Adapted with permission from [123], copyright 2020, American Chemical Society.

Different studies have been referring to the use of a magnetic mesoporous silica nanoparticle-based system to deliver anti-VEGF siRNA as antiangiogenic therapy. The system is assembled by loading the siRNAs into the M-MSNs, followed by capping the pores with polyethylenimine, grafting with polyethylene glycol (PEG) and modifying the surface with a fusogenic peptide (KALA) to enhance cellular uptake and endosomal release [120,121,122]. The systemic administration of this nanocarrier has shown effective tumor growth inhibition in both ectopic and orthotopic models of lung cancer, as well as remarkable retardation of tumor growth in orthotopic ovarian tumor-bearing mice, without systemic toxicity. Due to the magnetic core of the nanoparticles, in vivo tracking of the system can also be easily done by MRI, which revealed that the nanocarriers were able not only to accumulate at the primary tumor site but also at the metastatic sites. Biodistribution studies measuring Fe content revealed that 10% of the administered dose of particles accumulated in the tumor region one day after injection, and after 3 days, ~4% was still retained, becoming almost undetectable after 7 days. After 24 h, Fe was also found in liver, lung, spleen and kidney, increasing its content in the kidney at 7 days, due to degradation and renal clearance. Additionally, using the PEGylated M-MSNs as carriers for siRNA increased the genetic material bioavailability by 10-fold, by increasing its mean retention time from ~70 min to ~310 min, and greatly reducing its plasma clearance [121]. Despite the great improvement in pharmacokinetics and the targeting efficiency of the nanosystems, upon treatment interruption, tumor growth would resume, revealing that the therapy was not effective in eliminating all the tumor cells. Therefore, therapeutic strategies also incorporating anticancer drugs or different anticancer mechanisms should be considered [121,122]. A novel and innovative ultrasound responsive gene delivery system was recently developed using core-shell M-MSNs loading microbubbles (M-MSN@MBs) [123]. pDNA was loaded into the pores of the M-MSNs modified with PEI, which were then encapsulated in lipid microbubbles, forming a larger carrier structure composed of multiple M-MSNs with an air core, as presented in Figure 18a. The study results demonstrated that the final nanocarrier presented a great improvement in cytotoxicity when compared to the M-MSNs. Additionally, it also demonstrated magnetic responsiveness and was attracted to the tumor area upon application of a magnetic field. Data of in vivo testing in ovarian tumor-bearing mice confirmed that ultrasound-targeted microbubble destruction (UTMD) was not only able to release the loaded M-MSNs but also improved the pDNA transfection efficiency by opening the blood-tumor barrier and increasing the permeability of the cytoplasmic membrane (sonoporation) (Figure 18b) [123].

### 5.4. Other Silica-Based Nanocomposites

Interesting nanosystems have been recently reported in literature combining silica with a range of different organic and inorganic materials, integrating their complementary properties, and thus achieving novel multifunctional nanocomposites, sometimes with synergistic effects.

**Figure 19:**
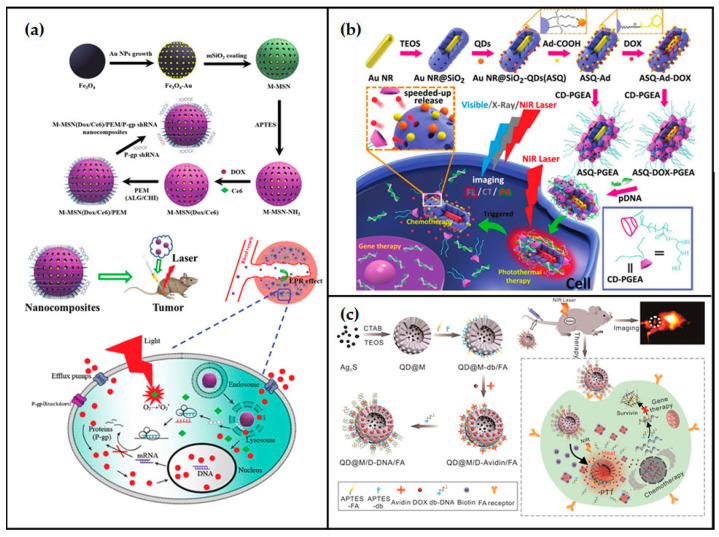
Illustration of silica-based nanocomposites for chemo/photo/gene therapy obtained by combining several different organic and inorganic materials: (**a**) Preparation of magnetic MSNs coated with alginate/chitosan polyelectrolyte multilayers and carrying DOX, Ce6 and P-gp shRNA, and their intracellular pH-triggered release in tumor cells (reproduced from [124], with permission from The Royal Society of Chemistry); (**b**,**c**) Preparation and working principle of multimodal imaging-guided therapeutic platforms ((**b**) adapted from [125], with permission from John Wiley and Sons; (**c**) reproduced from [126], with permission from Ivyspring International Publisher).

For instance, magnetic MSNs were synthesized with a core composed of Fe_3_O_4_–Au nanoparticles, which enabled dual-modal MR and tomography (CT) imaging, and both the photosensitizer chlorin e6 (Ce6) and DOX were loaded onto the aminated MSNs, as illustrated in Figure 19a [124]. Finally, alginate/chitosan polyelectrolyte multilayers were assembled on the surface of the nanoparticles, not only to serve as pH-responsive drug gatekeepers but also to enable the binding of shRNA against P-gp for reversing multidrug resistance. These nanocomposites presented an average diameter of 280 nm and their in vivo administration in tumor-bearing mice revealed significant tumor reduction, which was even further improved by laser irradiation, corroborating the synergistic effect of the different materials [124]. Shun and colleagues, on the other hand, described the use of a mesoporous silica layer as an intermediate between Au nanorods, used for CT and photoacoustic imaging, and fluorescent quantum dots (QDs), that enhance the imaging sensitivity [125]. Additionally, the mesoporous silica allowed the loading of DOX, which was then sealed by two-armed ethanolamine-modified poly(glycidyl methacrylate) with cyclodextrin cores (CD-PGEA), enabling pDNA complexation (Figure 19b). Interestingly, the photothermal effect of Au nanorods was used for photothermal therapy (PTT) and the resulting heat induced the release of DOX through the facilitated detachment of polycationic gatekeeper for controlled chemotherapy. The in vivo antitumor performance of the nanosystem was assessed in glioma tumor-bearing mice and a pDNA encoding the p53 antioncogene was used. The results of DOX- and p53 DNA-loaded nanoparticles administration and NIR irradiation revealed remarkable and continuous tumor inhibition, indicating the synergistic effects of combined photothermal/chemo/gene therapy [125]. More recently, mesoporous silica core-shell nanoparticles were developed encapsulating silver sulfide QDs and surface modified with desthiobiotin (db) and folic acid, for active tumor targeting [126]. DOX was loaded into the MSNs pores and avidin was applied as a drug gatekeeper via db-avidin interaction (Figure 19c). Based on that interaction, a db-modified antisense oligonucleotide (db-DNA) able to inhibit survivin expression, an inhibitor of apoptosis greatly expressed in tumor cells and associated with drug resistance, was also grafted to avidin. After receptor-mediated endocytosis, both DOX and db-DNA release was triggered by the overexpressed biotin present in cancer cells. In vitro and in vivo experiments showed that the nanosystem was biocompatible and presented targeting ability and fluorescence imaging ability, provided by the QDs, which were also useful for photothermal therapy. Once again, the synergistic effect of combined biotin-responsive drug and gene co-delivery, and PTT, resulted in significantly enhanced antitumor efficacy with minimal systemic toxicity, in HeLa tumor-bearing mice [126].

## 6. Cellular Uptake and Intracellular Fate of Silica-Based Vectors

The efficiency of silica-based gene delivery systems is strongly affected by their cellular uptake and intracellular fate. In turn, these processes are influenced by several physicochemical parameters of the carriers, such as size, shape, surface roughness and surface functional groups [127,128]. Despite several studies describing the influence of size and surface charge of silica particles on their cellular uptake [129,130,131], few are about silica particles as gene carriers. One of them was led by Yu and his research team [46], where they synthesized non-porous silica nanoparticles and modified them with amine groups at the surface. These particles had diameters between 125 and 570 nm and the plasmids DNA tested had 6.1 kbp, or 8.9 kbp.

They observed, in serum-free medium, that the cellular uptake in HEK 293T cells increased with the increase of the particles’ diameter, obtaining the highest value of internalization for 570 nm of diameter. They also observed that bare particles had higher cellular uptake performance when compared with particles/DNA complexes. This fact could be explained by the influence of the surface charge of the particles in the endocytic process since bare particles had a positive surface charge while complexes were negatively charged. Interestingly, they also observed that despite the higher uptake of complexes with bigger sizes, they presented lower GFP expression, showing that size also influences the intracellular path and/or the delivery of the pDNA into the nucleus. The highest transfection level, for both plasmids tested, was obtained for particles with a diameter of 330 nm. Another study showed that pore size also influences the cellular uptake mechanism of the MSNs-based complexes [132]. In this study, different MSNs with the same composition and surface charge and similar sizes (~200 nm) but distinct pore sizes (2, 4, 7, 10, and 23 nm) were synthesized. To evaluate the endocytic pathway used by these different MSNs diverse endocytosis inhibitors were added to the cells: chlorpromazine, a clathrin-dependent endocytosis inhibitor; genistein, a caveolae-dependent endocytosis inhibitor; and amiloride, a macropinocytosis inhibitor. The energy-dependent cellular uptake was also studied by incubating cells at 4 °C. Interestingly, the obtained results showed that pore size influences the cellular uptake of the MSN-based vectors, which could be due to the different surface roughness among the complexes tested. While complexes based on MSNs with a pore size of 2 nm were internalized mainly by caveolae-dependent endocytosis and macropinocytosis, the other MSNs complexes entered by caveolae- and clathrin-dependent endocytosis and showed to be energy-dependent In fact, the surface of the complexes has a great influence on transfection efficiency, since the interaction between cells and nanosystems depends not only on the surface roughness, but also on the chemical groups presented at their surface. The chemical groups will influence the type of interaction between the nanosystems and cells, either by their charge and/or by their possible interactions with specific membrane receptors [21]. Indeed, the surface modification of silica particles is one of the most used strategies to improve their performance as gene carriers [20].

In fact, conferring a positive surface charge to silica nanoparticle/DNA complexes is one of the most used modification strategies to improve cellular uptake. The increase of surface charge of complexes will lead to a higher cell membrane interaction, and, consequently a higher cell uptake. As explained in Section 1, the most explored ways to perform this modification are either using a silica precursor with a cationic group, such as APTES, or using a cationic polymer. Coating the MSN with a high density of cationic charges improves the cellular uptake, as demonstrated by the study on which MSNs were coated with branched PEI (M_w_ = 0.8 kDa) (MSN-PEI), which achieved a cellular uptake percentage above 92% in human dermal fibroblasts (HDFs) [133]. Another study using MSNs modified with the cationic polymer PEI (Mw ~1.2 or 25 kDa) showed a higher uptake (2 orders of magnitude) than non-modified ones or modified with the anionic 3-(trihydroxysilyl)propyl methylphosphonate or non-ionic charged PEG [70]. A different work with silica-based complexes that demonstrates the influence of the positive charge in the cellular uptake of these type of systems was developed for large pore MSN-grafted with PLL to deliver siRNA [134]. The authors showed, by confocal laser scanning microscopy, that this formulation enters HeLa cells to a higher extent than non-modified or amine-modified ones. This result was also confirmed by flow cytometry analysis, where complexes based on MSN-grafted with PLL resulted in ~61.6% of cellular internalization, in contrast with ~39.1% and 0.38% of amine-modified or non-modified MSN complexes, respectively. The authors hypothesized that interfacial interactions between the several positive charges of PLL and the negative surface charge of cells could be favorable. A study involving silica nanoparticles modified with APTES (MSN-NH_2_) or l-histidine (MSN-His), which is an amino acid positively charged at physiological pH, corroborates the importance of chemical groups at the surface in the cellular uptake of the nanocomplexes [135]. Through flow cytometry assays, it was shown that cells incubated during 2 h with complexes based on MSNs modified with l-histidine (5/1 and 15/1 N/P ratios) had an uptake above 80% in contrast with 20% and 40% of cellular uptake for MSNs modified with APTES at 5/1 and 15/1 N/P ratios, respectively. Additionally, the mean fluorescence intensity (MFI) of cells, for both modified MSNs, was almost 3-fold higher at 15/1 N/P ratio than at 5/1 ratio. This improvement of cell uptake by MSN-His could be explained by the higher quantity of amine groups at 15/1 N/P ratio and consequently higher positive charge to interact with the cell membrane. In addition, they showed, through monensin treatment, that both complexes were localized in non-acidic compartment after 2 h of incubation, demonstrating the ability of these complexes to escape the endolysosomal pathway. Moreover, they showed by fluorescence microscopy that, after 4 h of incubation, complexes based on MSN-NH_2_ were localized near the cell membrane while MSN-His/DNA complexes were localized closest to the nucleus. Concerning this last observation, several studies showed by different microscopy techniques that positive surface charged complexes based on silica nanoparticles were localized near [69,136,137,138] or inside [139] the nucleus, highlighting not only the stability of the complexes but also its ability to carry the genetic material to the nucleus. Another common observation is the increase of cellular uptake with the time of incubation of positively surface complexes with cells. For example, Li and co-authors observed that complexes based on MSN modified with ammonium salt resulted in a percentage of cellular uptake of 29.1%, 40.7% and 87.2% after 1 h, 2 h, and 4 h of incubation with cells, respectively [137].

Another strategy to improve the cellular uptake of the complexes is to functionalize them with molecules that act as specific ligands to promote a receptor-mediated endocytosis. For instance, the internalization mechanism for a system composed by magnetic MSNs coated with folic acid-conjugated polyethylenimine (FA-PEI) (M-MSN/PEI-FA) to co-deliver doxorubicin and shRNA was studied by Li et al. [140]. It was observed by confocal laser scanning microscopy that, after 4 h of incubation, these hybrid nanosystems were much more internalized by HeLa cells than nanosystems without PEI-FA coating or M-MSN/PEI-FA incubated with cells previously treated with free folic acid, confirming that the cellular uptake of these nanosystems is mediated by a ligand-receptor interaction. To clarify the cellular uptake mechanism involved, the authors incubated HeLa cells 30 min prior to the addition of the nanosystems with inhibitors for different endocytic pathways: chlorpromazine, genistein, nystatin (lipid raft-caveolae endocytosis pathway inhibitor), cytochalasin D (macropinocytosis inhibitor), or sodium azide at 4 °C (inhibitor of cytochrome c oxidase (an ATP-synthase inhibitor). The observed results showed that nanosystems were internalized by clathrin-mediated endocytosis and that was an energy-dependent process. Another study using complexes based on MSNs grafted with mannosylated polyethylenimine showed specificity to mannose receptors in cells with abundant mannose receptors (Raw 264.7) [141]. Complexes containing different mol% of mannose groups, from 4.17 until 15.9 mol%, were assessed. These formulations were assessed in the presence of free mannose as a competitor for the complexes. The obtained data showed an inhibition of the transfection activity for complexes based on modified MSNs with 7 mol% or higher of mannose groups. The minimum amount of ligand for receptor-mediated gene delivery was also reported for other galactose- or mannose-based targeting complexes [142].

**Figure 20:**
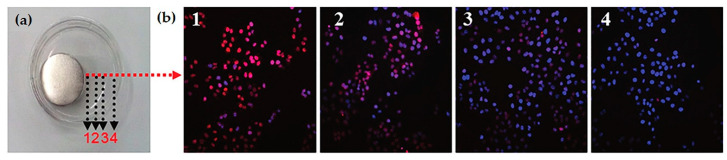
Photography of the experimental setup (**a**) and respective confocal images of MCF-7 cells (**b**) after 12 h of incubation with the systems based on magnetic MSNs, where B1 is the image closest to the magnet and B4 is the farthest one. Nuclei were stained with DAPI (blue color) and systems contain doxorubicin with fluorescence properties (red color). Reproduced from [124], with permission from The Royal Society of Chemistry.

As mentioned above, shape is another important physicochemical property that influences the cellular uptake and the intracellular path of the silica-based vectors. Wang and co-authors studied, by confocal fluorescence microscopy, the effect of the shape of magnetic MSNs modified with a copolymer of poly(l-lysine) and poly(ethylene glycol) (M-MSN-PLL-*g*-PEG) in the cellular uptake of the complexes [116]. For this purpose, they synthesized rod-like and sphere-like hybrid MSNs to carry pDNA. They observed that despite an enhanced cellular uptake when an external magnetic field was applied and a co-localization in lysosomal compartment of the cells for both formulations, the rod-shaped magnetic MSN-PLL-*g*-PEG complexes showed the highest mean fluorescence intensity (MFI), either in the presence or absence of an external magnetic field. Indeed, the use of magnetic-responsiveness properties (such as, incorporating iron oxide nanoparticles) has been also explored for the enhancement of the cellular uptake of the silica-based complexes [74,143,144]. An interesting cellular uptake study using magnetic-responsive MSNs-based vectors was developed by Yang and colleagues using magnetic MSNs coated with polyelectrolyte multilayers of alginate and chitosan to co-deliver doxorubicin and shRNA [124]. They observed a differentiated cellular uptake according to the distance of the MCF-7 cells to an external magnetic field (Figure 20).

The closer the cells were to the 0.42 T magnet, the higher was the cell uptake, showing the magnetic responsiveness of these nanosystems. The same research group observed a similar result in other work also involving hybrid magnetic MSNs [140].

These studies corroborate that the design of an efficient silica-based gene delivery system is a laborious and meticulous process that should take into account several parameters in order to achieve the intended objective.

## 7. Biocompatibility of Silica-Based Materials

The safety and biocompatibility of any gene delivery system, or pharmaceutical product in general, are matters of the uppermost concern and are presented as a prerequisite before any type of clinical testing, to ascertain that undesired adverse effects do not occur. Regarding silica-based nanosystems, multiple investigations have been carried out to assess their harmful effects, and despite their overall safety being supported, variable results have been yield in different studies, namely concerning the correlation of toxicity with different physicochemical properties of the system, as detailed in various recent reviews [145,146,147].

The cytotoxicity associated with silica nanosystems has been described to be extremely dependent on their surface chemistry, being mainly attributed to two factors: (1) the density of surface silanol groups, which can interact with cell membrane components leading to membranolysis; and (2) the generation of silica reactive oxygen species (ROS) [146,148,149]. Indirectly, these factors are related with other physicochemical properties of the nanosystems, such as size and porosity, which affect the surface area. For instance, Kim and colleagues studied the biological activity of monodisperse silica nanoparticles with diameters ranging from 2–200 nm in A549, HepG2, and NIH/3T3 cells and concluded that the toxicity of the nanoparticles is size, dose, and cell type dependent [150]. Despite the variable results obtained with the different cell lines, the authors concluded that, in general, a larger particle size leads to higher cytotoxicity at any given concentration after 24 h of exposure; however, these effects seemed to be reduced after 72 h [150]. These finds, however, were not in agreement with several other studies, in which it was concluded that smaller nanoparticles presented higher cytotoxicity, due to the higher availability of silanol groups [145,151,152]. Additionally, the data collected in a recent systematic review on the toxicity of silica nanoparticles, on which scientific papers from 2010 to 2016 were thoroughly assessed, showed that the smaller the size, the stronger the pro-inflammatory effect of silica nanoparticles [153]. Additionally, in this systematic revision, it was noted that regardless of the cell line or incubation period, significant cytotoxic effects were only observed at nanoparticle concentrations of 25 µg/mL or above. Several different studies have also reported this inappreciable cytotoxicity of silica NPs and MSNs at low doses (<50 μg/mL) [149,152,154], with some reporting high cell viability even at higher doses of NPs. For instance, Li and colleagues, evaluated in vitro toxicity of silica NPs to lung cells and found that in 16HBE cells significant cytotoxicity was induced only at a dosage of 128 μg/mL; however that same dosage did not present a toxic effect in A5490 cells [155]. On the other hand, Chauhan and co-workers recently highlighted MSNs as safe nanoparticles for drug delivery. In their study, the cytotoxicity of MSNs with approximately 123 nm was assessed in bone marrow mononuclear cells and SHSY5Y neuronal cells, and they found that at low doses (1–10 μg/mL for SHSY5Y cells and 5–25 μg/mL for BM-MNCs) MSNs were nontoxic [156].

As mentioned before, the surface properties and porosity are also crucial factors influencing biocompatibility. Lehman and colleagues compared in vitro cellular toxicity of both amine-functionalized and non-functionalized mesoporous and nonporous silica nanoparticles in a murine macrophage cell line (RAW 264.7), and found that mesoporous materials were less toxic that nonporous materials, and that surface modification was able to reduce toxicity by reducing free radical production [149]. These results were in agreement with other reports of mesoporous silica exhibiting lower hemolytic effect compared to nonporous silica, which has been explained by the lower density of silanol groups on the surface due to the presence of mesopores [56,146]. Therefore, as mentioned before, altering the surface of silica-based nanosystems, namely by PEGylation, has been a widely used and successful method to improve biocompatibility and pharmacokinetics of these nanosystems. Pisani and co-workers, for instance, assessed the biocompatibility of magnetic mesoporous silica nanoparticles, with different surface modifications, in human HepaRG cells. Pristine, PEGylated, and lipid (DMPC)-coated magnetic MSNs were evaluated, and neither were extremely harmful to liver cells, having the authors set a limit of biocompatibility of 60 μg/mL of these NPs for these cells. The maximum decrease in cell viability corresponded to 35% and was reached by 48 h of exposure to 400 μg/mL of pristine NPs, which also demonstrated the improvement in biocompatibility achieved with the surface modifications [114].

Regarding genotoxicity of silica-based nanosystems, it has not been as extensively investigated as their cytotoxicity, but recent studies have raised some concerns. According to data from the systematic revision by Murugadoss and colleagues, both colloidal and Stobër silica NPs revealed genotoxic effects, mainly associated with oxidative stress induction, in human tumor cell lines from lung, kidney, skin and gastro-intestinal system. Especially in skin-derived cell lines, DNA strand breaks were observed at low concentrations (2.5–10 µg/mL) [153]. Additionally, it has been reported that MSNs caused genotoxicity to normal human cells, namely human embryonic kidney 293 (HEK293) cells [157]. The results of this study have shown that, when treated overnight with MSNs at a concentration of 120 µg/mL, HEK293 cells presented up- or downregulated expression of certain genes and DNA degradation [157]. Královec and colleagues evaluated the cytotoxic and genotoxic potential of silica-coated iron-oxide nanoparticles (IONPs) in human HK-2 renal proximal tubule epithelial cells and reported severe disruption of the microtubule cytoskeleton structure, which resulted in antiproliferative and cytotoxic effects [158]. In this study, genotoxic effects were also described after short term exposure to 25 and 100 μg/mL of silica-coated IONPs [158]. However, different studies have also reported very limited genotoxicity of silica nanoparticles, being this a controversial subject [152,159]. A recent systematic review regarding the genotoxicity of amorphous silica nanoparticles highlighted the abundance of conflicting reports and the need to conduct further investigation in a more systematic manner. According to the authors, the genotoxicity of these nanoparticles is highly dependent on two major classes of parameters: the physicochemical properties of the nanosystem (such as size, charge, porosity and surface area) and the treatment conditions (such as dosage, exposure time, cell type, animal model, etc.) [160].

In vivo toxicity has been evaluated using rats and mice and various different administration routes, which have been proved to influence the absorption, biodistribution and toxicity of the nanosystems. Overall, short-term exposure to silica nanoparticles via intravenous route showed some sign of damage/toxicity. Some studies have shown accumulation of silica nanoparticles in organs such as the liver or spleen; however, that was not generally associated with any major effects [146,153]. Upon all these controversial results, the FDA approval of hybrid silica NPs for imaging purposes marked an extremely important step in supporting the clinical viability of silica-based nanosystems, as already mentioned in Section 1. The first human clinical trial with C-dots has indicated the safety of those silica-based nanoparticles for human use, having reported no toxic or adverse effects related with the particles [14]. Additionally, a clinical trial with silica–gold NPs for atheroprotective management of plaques evaluated two different delivery techniques for the NPs and both approaches presented an acceptable level of safety for clinical practice [161]. On the other hand, after promising preclinical results, Bukara and colleagues also conducted a clinical trial assessing the potential of ordered mesoporous silica nanoparticles (OMS) in enhancing the bioavailability of fenofibrate in man. In this study, fenofibrate-loaded OMS were enclosed within capsules and administered to the volunteers and compared with administration of a marketed formulation of fenofibrate. Besides revealing enhanced results in the absorption of fenofibrate, the OMS formulation was safe and well tolerated by the volunteers, which demonstrated no clinically relevant changes in any electrocardiogram parameter or vital signs [161].

## 8. Silica-Based Systems as an Alternative to Other Vectors

As mentioned before, due to their versatility, safety, and stability, silica-based gene delivery vectors have been emerging as a promising alternative to other types of currently used vectors, such as polymers, lipids, or even viral vectors. Herein, in order to better compare and illustrate the advantages of silica-based systems in terms of safety and performance, Table 4 summarizes information presented in studies mentioned above, on which there is a direct comparison between different types of silica-based vectors and other types of more commonly used vectors.

As previously mentioned, PEI 25 kDa is generally considered the gold standard for polymeric gene delivery due to its high transfection efficiency. However, as described in Table 4, silica-based vectors coated with smaller molecular weight PEI [23] or with different polymers [82] were able to achieve similar results in transfection, while being much safer. Generally, the toxicity of polycations is greatly decreased when they are surface-adsorbed to silica nanoparticles or complexed with DNA, when compared to the toxicity of polymers in solution, since the surface immobilization and complexation reduce the polymer chain flexibility and charge density [68,74]. Additionally, simply aminated silica structures, presented better overall results than PEI or polymer-based commercial transfection agents [31,40].

Regarding lipid-based gene delivery systems, several studies reported similar or even better performance results in vitro using positively charged silica nanosystems when compared to widely used lipid-based transfection agents, such as Lipofectin^TM^, Lipofectamine^®^ or Oligofectamine^TM^, which have excellent transfection efficiencies [44,47,54,55,71,115]. Additionally, the silica-based systems proved to be much less toxic in vitro and presented in vivo safety, contrary to the transfection reagents, which are not applicable in vivo. When it comes to comparing protocells (liposome-coated MSNs) with the corresponding liposomes, protocells presented significant advantages, namely, much higher cargo capacity, improved stability, superior efficiency, and mitigated toxicity [105,106].

Despite the lack of information available concerning a direct comparison between silica-based vectors and viral vectors, it is known that the later are associated with major drawbacks, mainly related with safety. As presented on the table above, a research group has reported amine-coated silica nanoparticles to be as efficient in transfecting genetic material as a viral vector, while causing no toxic effects [42]. Moreover, Sapre and colleagues have recently reported the cloaking of adenovirus (Ad) with silica, as a way of enhancing gene delivery while reducing immunogenicity. In this work, silica-coated Ad enhanced tumor transduction by 23-fold while reducing liver uptake and off-tumor targeting by 210-fold, which minimized hepatotoxicity [162].

## 9. Conclusions

Gene therapy has provided a new and promising strategy for the effective treatment of diseases involving genetic malfunctioning by correcting them at the gene expression level, having the potential to change current medicine’s paradigm. Therefore, the search for ideal carriers for gene delivery has been an area of great interest to the research community since it is a crucial factor for the therapeutic success of gene therapy. Despite their popularity, viral vectors present some fundamental drawbacks that can be overthrown by nonviral vectors, which have increasingly received more attention in the last few decades, mainly due to their higher safety.

In the present review, the current progress in silica-based vectors for gene delivery has been reported, highlighting the diversity of structures and versatility of this material. Thanks to the remarkable biocompatibility of silica and its FDA recognition, several different types of silica-based structures have been recently reported for gene therapy applications, including both forward transfection (particulate systems) and reverse transfection methods (silica sheets), and even as sedimentation agents (non-porous silica nanoparticles). Additionally, the easily adaptable silane chemistry grants the silica-based nanosystems the ability to have controlled release properties by introducing stimuli-responsive groups both in the framework or at the surface, which can also be simply modified by taking advantage of electrostatic interactions with cationic materials.

From the range of reported structures, mesoporous silica nanoparticles are undoubtedly the most prominent nanosystems due to their numerous useful and attractive properties as carriers, such as high surface area and a large surface-to-volume ratio, which confer them a great loading capacity. MSNs have been combined with lipidic coatings and more commonly with cationic polymers and specific targeting ligands, resulting in nanosystems with increased performance in terms of DNA binding, protection, and transfection efficiency when compared to non-modified or aminated MSNs. Notably, the combination of MSNs with these materials also presents an improvement in comparison to the use of the polymers or lipids alone, resulting in a decrease of toxicity of cationic polymers and an increase in the loading capacity, stability, and bio-applicability of traditional liposomes. Furthermore, interesting combinations with magnetic nanoparticles, or even gold or quantum dots, have opened the possibility to the development of multifunctional MSNs as theranostic platforms, which can improve the outcome of gene therapy by combining it with imaging functions, as well as magnetically guided delivery and both photothermal and chemotherapy.

Despite the current lack of clinical trials regarding gene therapy applications of silica-based vectors, clinical trials on the imaging and phototherapeutic applications of silica nanoparticles and silica-gold nanoparticles have been occurring, which highlights the promising clinical viability of these nanosystems. Additionally, as presented in this review, promising therapeutic applications of silica-based gene delivery systems have already been vastly studied not only in vitro but also in vivo, having diverse antitumor strategies been reported in several different cancer models. For instance, MSNs coated with PLL, or polymerized dopamine were used to carry therapeutic genes and presented interesting results in the transdermal delivery to the treatment of SCC of the skin, and in the treatment of colorectal cancer, respectively. Additionally, CD-PGEA coated and disulfide-based MSNs were successfully used to co-deliver therapeutic drugs and genes in glioma treatment. PEI and PEG-coated magnetic MSNs, modified with a fusogenic peptide, have been employed to deliver siRNAs for antiangiogenic therapy in both models of lung and ovarian cancers, revealing no systemic toxicity and effective specific accumulation at the primary tumor and metastatic sites. PEGylated lipid-coated MSNs have also recently been described for combined gene and chemotherapy to HCC and orthotopic breast cancer, revealing synergistic antitumor and antimetastatic effects and significant suppression of the primary tumor growth. On the other hand, other therapeutic applications, such as brain or spinal cord therapies, have been described using silica-based vectors. Amine-modified MSNs were reported for co-delivery of genetic material for brain therapies, while lipid-coated MSNs (protocells) were used as carriers for an anti-inflammatory and pain-suppressive therapeutic transgene to the spinal cord.

In conclusion, the inherent safety and biocompatibility of silica, combined with the vastly explored potential of silica-based nanosystems have revealed very promising results for future clinical application, being these nanosystems advantageous alternatives to viral vectors and even to other commonly used polymeric or lipidic vectors.

## Figures and Tables

**Table 1:** Clinical trials on silica-based nanoparticulate systems.

Name	Particle Type	Application	Study Number	Submission Date	Status
C-Dots	Silica NPS with a NIR fluorophore, PEG coating, and a ^124^I-labeled cRGDY targeting peptide	PET and fluorescent imaging of melanoma and malignant brain tumors	NCT01266096	24 December 2010	Active, not recruiting
			NCT02106598	8 April 2014	Recruiting
			NCT03465618	14 March 2018	Recruiting
	Silica NPS with a NIR fluorophore, PEG coating, and a ^64^Cu-labeled PSMA-targeting particle tracer	Image-Guided Surgical Treatment of Prostate Cancer	NCT04167969	19 November 2019	Recruiting
AuroLase^®^	PEG-coated silica-gold nanoshells for NIR facilitated thermal ablation	Thermal ablation of solid primary and/or metastatic lung tumors	NCT00848042	20 February 2009	Completed
			NCT01679470	6 September 2012	Terminated
			NCT02680535	11 February 2016	Active, not recruiting
n.d.	Silica-gold NPS and iron-bearing silica-gold NPS	Plasmonic photothermal therapy (PPTT) of atherosclerotic lesions	NCT01270139	5 January 2011	Completed

“n.d.”: not defined; “NPs”: nanoparticles; “PEG”: poly(ethylene glycol) “NIR”: near-infrared.

**Table 2:** Examples of different approaches reported for the obtention of large-pore sized MSNs.

Size (nm)	Pore-Enlarging Method	Surface Modification	Ref.
**MSNs: 250** **Pores: 23**	Swelling agent: TMB	APTES	[51]
**MSNs: 200–400** **Pores: 9**	Co-solvent: ethanol Template: mixed surfactants (fluorocarbon + hydrocarbon)	Octadecyl group	[54]
**MSNs: 70–300** **Pores: 20**	Swelling agent: TMB Template: mixed surfactants (fluorocarbon (FC-4) + Pluronic F127) Low synthesis temperature and subsequent hydrothermal treatment	APTMS	[52]
**MSNs: 200–300** **Pores: ~15**	Template: PS-*b*-PAA Stabilizing agent: CTAB surfactant	APTES	[55]
**MSNs: ~400** **Pores: 24**	Hydrothermal treatment: stability difference-based selective bond breakage	APTES + PβAE	[53]

“TMB”: 1,3,5-trimethlybenzene; “APTES”: (3-aminopropyl)triethoxysilane; “APTMS”: (3-aminopropyl)trimethoxysilane; “PS-*b*-PAA”: Polystyrene-*b*-poly(acrylic acid); “CTAB”: cetyltrimethylammonium bromide; “PβAE”: Poly(β-amino ester).

**Table 3:** Examples of gene delivery systems based on polymer-modified silica nanoparticles.

Silica Nanoparticle Framework	Polymeric Surface Modifying Agent	Payload	Target Gene	Tested Cell Type	In Vivo Testing	Ref.
MSNs smaller than 130 nm and pore size of ~2.5 nm	PEI (1.3 kDa)	siRNA	eGFP, Akt, K-ras	PANC-1	-	[66]
Phosphonated MSNs with d ~165 nm	PEI (10 and 25 kDa)	CQ and pDNA	GFP	Neuro-2A	-	[67]
Polyamine-functionalized silica NPs of ~10 nm	PEI (1.8, 10 and 25 kDa)	pDNA	pEGFP-N1	Neuro-2A	-	[68]
Amine-functionalized MSNs of ~100 nm with average pore size of 4.58 nm	PEI (25 kDa)	pDNA	pEGFP-C1	293T and A549	-	[69]
MSNs with 100–130 nm diameter and pore size of ~2.5 nm	PEI (0.6 to 25 kDa)	siRNA, pDNA and paclitaxel	GFP	PANC-1, BxPC3, RAW 264.7, BEAS-2B and HEPA-1	Mice	[70]
MSNs with 47 nm diameter	PEI (1.8 and 10 kDa) + PEG	siRNA and antibody (trastuzumab)	Luc, HER2	HER2+ (MDA-MB-31-H2N-luc, BT474, SKBR3, HCC1954, and JIMT-1) and HER2^−^ (MCF-7, MDA-MB-231, MDA-MB-468, MCF-10a, HepG2, HEK-293)	Orthotopic HCC1954 tumor-bearing SCID mice	[65,73]
Amino-functionalized MSNs of 70 nm and pore size ~4.8 nm	PEI + hbPEI (1.3 kDa)	siRNA and oligonucleotides	GFP	MDA-MB-231	-	[71,72]
MSNs with an average diameter of 56.7 nm and pore size of 2–3 nm	PEG + PDMAEMA or PEG + PDEAEMA	CQ and pDNA or siRNA	Luc, GAPDH	B16F10	-	[74]
Solid and hollow NPs ~100 nm; Nanorods with d~100 nm and l~300 nm; Chiral nanorods d~100 nm and l~200 nm or ~300 nm	PDMAEMA	pDNA	pRL-CMV, pEGFP-N1	COS-7 and HEPG2	-	[75]
Disulfide-bridged silica nanoparticles with incorporated DOX	CD-PGEA	pDNA and DOX	pRL-CMV, pEGFP, p53	HepG2 and C6	C6 glioma tumor- bearing BALB/c nude mice	[60]
Amine-functionalized MSNs of ~80 nm	PAMAM dendrimer conjugated chitosan	pDNA and DOX	p53	Hela	-	[76]
Hollow MSNs with ultra-large pores of ~24 nm	Poly(β-amino ester)	siRNA and DOX	P-gp	MCF-7/ADR	MCF-7/ADR tumor-bearing BALB/c nude mice	[53]
Azide-functionalized MSNs of 35–60 nm with 2.6 nm pore size	Poly l-arginine	pDNA and DOX	mCherry	HeLa and A549	-	[77]
MSNs of ~200 nm with 4 nm pore size	Poly l-lysine	siRNA, oligonucleotides	GAPDH, CTGF, TGFβR-1	RT3	SCID mice	[78]
MSNs 100–200 nm with pore sizes of 4–5 nm: internally lined with amino groups and externally with mercapto groups or disulfide-bridged amino groups	Block copolymer containing cationic artificial amino acids and oleic acid blocks	siRNA	eGFP-Luc	KB, MCF-7	-	[81]
Amine-functionalized silica NPs with 90 nm	Hyaluronic acid	siRNA and pDNA	GFP	A549	Tumor-bearing BALB/c nude mice	[82]
Amine-functionalized MSNs of ~140 nm	Hyaluronic acid	siRNA and TH287	MDR1	CAL27	Tumor-bearing BALB/c nude mice	[83]
Amine-functionalized MSNs of ~125 nm	Polymerized dopamine	Anti-miRNA and AS1411 aptamer	miR-155	Human CRC (SW480, HT-29, SW620, Lovo, and Caco-2) and NCM460	SW480 tumor-bearing BALB/c nude mice	[84]

“CQ”: Chloroquine; “DOX”: Doxorubicin; “MSNs”: Mesoporous Silica Nanoparticles; “PEI”: poly(ethyleneimine); “hbPEI”: hyper-branched PEI; “PEG”: poly(ethylene glycol); “PDMAEMA”: poly(2-(dimethylamino)ethyl methacrylate); “PDEAEMA”: poly(2-(diethylamino)ethyl methacrylate); “CD-PGEA”: β-cyclodextrin core and two ethanolamine-functionalized poly(glycidyl methacrylate) arms; “PAMAM”: poly(amidoamine); “CRC”: colorectal cancer”.

**Table 4:** Comparative studies between silica-based systems and other vectors.

Silica-Based Vector	Other Vectors		Main Comparative Results		Ref.
			Performance	Safety	
Nanotubes functionalized with bPEI (1.8 kDa)	Polymers	bPEI (1.8 or 25 kDa)	In both HeLa and NIH3T3 cells, at N/P = 10, bPEI-nanotubes showed: ~500 times higher transfection efficiency than bPEI 1.8 kDa. comparable efficiency to bPEI 25 kDa. Bare nanotubes showed higher transfection efficiency than bPEI 1.8 kDa.	In both HeLa and NIH3T3 cells: bPEI-coated nanotubes and bPEI 1.8 kDa exhibited much higher cell viability than bPEI 25 kDa at all the tested N/P ratios At a N/P ratio of 20, bPEI-coated nanotubes resulted in greater cell viability than bPEI 1.8 kDa	[23]
Amine-coated solid NPs		PEI (60 kDa)	Silica NPs reached 30% of the transfection efficiency achieved with PEI in Cos-1 cells.	At concentrations used for transfection, the use of PEI led to 50% reduction in cell viability, while no significant toxicity was detected using silica NPs.	[40]
HA-coated solid NPs		PEI (25 kDa)	The gene silencing effect observed with silica NPs was efficient and similar to the effect obtained with PEI.	On A549 cells, silica NPs presented low cytotoxicity (<20%) even at 128 μg/mL; PEI presented an IC50 value of about 8 μg/mL.	[82]
Amine-coated upright nanosheets		PolyFect (activated dendrimers)	Transfection efficiency in hMSCs was ~85% for both.	Cell viability in hMSCs was 80–90% without PolyFect and 3% with PolyFect.	[31]
MSN-hbPEI	Peptide	Peptide-based vector L1 with ligand and PEI 25 kDa	The transfection using PEI-coated MSNs reached more than 2-fold GFP intensity (%) in MDA-MB-231 cells than that using both vector L1 and PEI.	Even at the higher concentration of 41 µg/mL, the nanoparticles did not exhibit any hemolytic activity. Vector L1 and PEI were not tested.	[72]
Amine-coated solid NPs	Lipids	Lipofectin^TM^	Silica NPs showed higher transfection efficiency than Lipofectin^TM^ in MCF-7 cells. When the ratio of silica:DNA was 9:1, GFP expression was 2-fold higher than with Lipofectin^TM^.	No tissue or cellular damages were observed in the mice injected with the pDNA/silica nanoparticles.	[44]
Positively charged MSNs		Lipofectamine® 2000	MSNs were more effective than Lipofectamine 2000 in Rex1 knockdown.	On iPSCs: Lipofectamine 2000 caused 43% of cell death at 96 h and 70% at 14th day; MSNs caused only 12% of cell death at 96th h and 30% 14th day.	[47]
Amino-coated large-pore MSNs		Lipofectamine® 2000	No significant differences were observed between the transfection efficiencies of MSNs and Lipofectamine® 2000 in SMMC-7721, HepG2 and Huh7 cells.	MSNs caused almost no toxic effects to SMMC-7221 cells at concentrations lower than 400 µg/mL in both 24 and 72 h incubations. Lipofectamine® 2000 was not tested.	[55]
Octadecyl-coated large-pore MSNs		Oligofectamine^TM^	The delivery efficiency of siRNA was not significantly different between MSNs and Oligo and was dose-dependent in both cases.	HCT116 cells showed negligible cytotoxic effect up to 40 μg/mL for both coated and uncoated MSNs. Oligo was not tested.	[54]
PEI-coated large pore magnetic MSNs		Oligofectamine^TM^	The delivery of siRNA to KHOS cells by MSN-based carrier led to a cell viability inhibition of 80%, while the same dose of siRNA delivered by Oligo resulted in only 50% cell viability reduction.	Both MSN-based carriers and Oligofectamine caused no significant cytotoxicity to KHOS cells.	[115]
MSN-hbPEI		Lipofectamine RNAiMax	Transfection with MSNs showed a time-dependent reduction in cell viability similar to Lipofectamine. There was no significant difference between their efficacies.	MSN-based carriers did not contribute to any significant toxicity to MDA-MB-231 cells after 72 h of incubation in concentrations of 2–50 µg/mL. Lipofectamine was not tested.	[71]
DOPC Protocell		DOPC and DOTAP liposomes	siRNA loading capacities of DOPC protocells, normalized against particle volume, are 50- and 10-fold higher than those of DOPC and DOTAP liposomes, respectively. Liposomes rapidly released their siRNA cargo under both neutral and acidic conditions. Protocells retained 95% of their encapsulated siRNA when exposed to SBF for 72 h. 300-fold less protocells than DOTAP liposomes were required to repress cyclin A2 expression by 90% in Hep3B cells.	DOPC protocells revealed much lower cytotoxicity than DOTAP liposomes in Hep3B cells.	[106]
Amine-coated solid NPs	Viral	HSV-1	Silica NPs transfection efficiency was equal or higher than that obtained using HSV-1 viral vectors.	Tissue damage was observed during intra-brain gene transfer using the viral vector, while no toxic effects were observed in mice treated with silica NPs even 4 weeks after transfection.	[42]

“MSNs”: Mesoporous Silica Nanoparticles; “PEI”: poly(ethyleneimine); “bPEI”: branched PEI; “hbPEI”: hyper-branched PEI; “HA”: hyaluronic acid; “Oligo”: Oligofectamine^TM^; “DOPC”: 1,2-dioleoyl-sn-glycero-3-phosphocholine; “DOTAP”: 1,2-dioleoyl-3-trimethylammonium-propane.
